# Spatiotemporal transcriptomic changes of human ovarian aging and the regulatory role of FOXP1

**DOI:** 10.1038/s43587-024-00607-1

**Published:** 2024-04-09

**Authors:** Meng Wu, Weicheng Tang, Ying Chen, Liru Xue, Jun Dai, Yan Li, Xiaoran Zhu, Chuqing Wu, Jiaqiang Xiong, Jinjin Zhang, Tong Wu, Su Zhou, Dan Chen, Chaoyang Sun, Jing Yu, Hongyi Li, Yican Guo, Yibao Huang, Qingqing Zhu, Simin Wei, Ziliang Zhou, Mingfu Wu, Ya Li, Tao Xiang, Huiying Qiao, Shixuan Wang

**Affiliations:** 1 Department of Obstetrics and Gynecology, Tongji Hospital, Tongji Medical College, Huazhong University of Science and Technology, Wuhan, China; 2 National Clinical Research Center for Obstetrical and Gynecological Diseases, Wuhan, China; 3 Key Laboratory of Cancer Invasion and Metastasis, Ministry of Education, Wuhan, China; 4 Department of Obstetrics and Gynecology, Zhongnan Hospital, Wuhan University, Wuhan, China; 5 Shanghai Health Commission Key Lab of Artificial Intelligence (AI)-Based Management of Inflammation and Chronic Diseases, Sino-French Cooperative Central Lab, Shanghai Pudong Gongli Hospital, Secondary Military Medical University, Shanghai, China; 6 College of Environmental and Resource Sciences, Zhejiang University, Hangzhou, China; 7 College of Plant Science and Technology, Huazhong Agricultural University, Wuhan, China; 8 Shanghai Ocean University, Shanghai, China

**Keywords:** Senescence, Reproductive disorders

## Abstract

Limited understanding exists regarding how aging impacts the cellular and molecular aspects of the human ovary. This study combines single-cell RNA sequencing and spatial transcriptomics to systematically characterize human ovarian aging. Spatiotemporal molecular signatures of the eight types of ovarian cells during aging are observed. An analysis of age-associated changes in gene expression reveals that DNA damage response may be a key biological pathway in oocyte aging. Three granulosa cells subtypes and five theca and stromal cells subtypes, as well as their spatiotemporal transcriptomics changes during aging, are identified. FOXP1 emerges as a regulator of ovarian aging, declining with age and inhibiting CDKN1A transcription. Silencing FOXP1 results in premature ovarian insufficiency in mice. These findings offer a comprehensive understanding of spatiotemporal variability in human ovarian aging, aiding the prioritization of potential diagnostic biomarkers and therapeutic strategies.

## Abstract (web summary)

Ovarian aging has an important role in health and fertility; however, the molecular mechanisms underlying it remain incompletely understood. Here the authors use single-cell and spatial transcriptomics in reproductively young, middle-aged and older human ovarian tissue to elucidate ovarian aging. They describe spatiotemporal changes in ovarian cells and highlight the important regulatory role of FOXP1.

## Main

Ovaries with the functions of fertility and hormone secretion play a vital role throughout the female reproductive lifespan^1^. Ovarian function peaks at approximately 20 to 30 years of age, begins to decline after 30 years of age, and then reaches failure at approximately 50 years of age^2^. People experiencing menopause will encounter a number of dysfunctional outcomes for approximately 30 years, such as osteoporosis, cardiovascular disease, obesity, tumors, Alzheimer’s disease and diabetes^3^. With the increase in life expectancy worldwide, ovarian aging has gradually become a key health problem among female persons undergoing menopause.

The development of therapeutic strategies to delay ovarian aging requires comprehensive understanding of the cellular components, molecular properties and their spatial–temporal changes. Human ovaries consist of different stages of follicles as basic functional units and a large number of stromal cellular elements^4^. Single-cell RNA sequencing (scRNA-seq), which is used to investigate cellular heterogeneity has facilitated the mapping of organ aging at unprecedented resolution^5^. Recent studies using scRNA-seq have indicated abundant cell types in human ovaries, such as granulosa cells, oocytes, stromal cells and immune cells^6,7^. Furthermore, studies on murine and primate ovaries have provided cell-specific findings during aging^8–14^; however, the diverse cellular landscapes and cell type-specific regulatory changes of human ovarian aging are still unknown.

The human ovary shows extensive variation in the cortex and medulla, with the processes of oogenesis starting in the cortex and the medullary region of the ovary undergoing dramatic restructuring. Thus, an understanding of spatial archetypes is necessary for a comprehensive understanding of the aging dynamics of the ovary. scRNA-seq technology requires the dissociation of tissue into a single-cell suspension, increasing the difficulty of studying spatial architecture of the ovary. Although some in situ hybridization (ISH)-based methods have obtained spatial information, they only detect a few known target genes simultaneously^15,16^. Spatial transcriptomics (ST) is a new technology that captures the messenger RNA of cells in sections using numerous barcoded oligo-dT primers and then maps transcripts to the tissue slice, enabling the spatial visualization of gene expression^17^. This approach facilitates an analysis with subcellular resolution to confirm regional markers and cell type identities based on ST and scRNA-seq.

In this study, we aim to explore the cellular spatiotemporal changes and key regulatory genes involved in human ovarian aging. We present a spatiotemporal atlas that systematically describes the spatial archetypes and cellular heterogeneity during the aging of the human ovary at three representative stages during ovarian lifespan: reproductively young (18–28 years), middle-aged (36–39 years) and older aged (47–49 years). Then, we identified FOXP1 as a central protective factor for ovarian aging. Our data provide valuable inspiration for the mechanism and potential therapeutic targets of human ovarian aging.

## Results

### Study design for spatiotemporal analysis of human ovarian aging

**Fig. 1:**
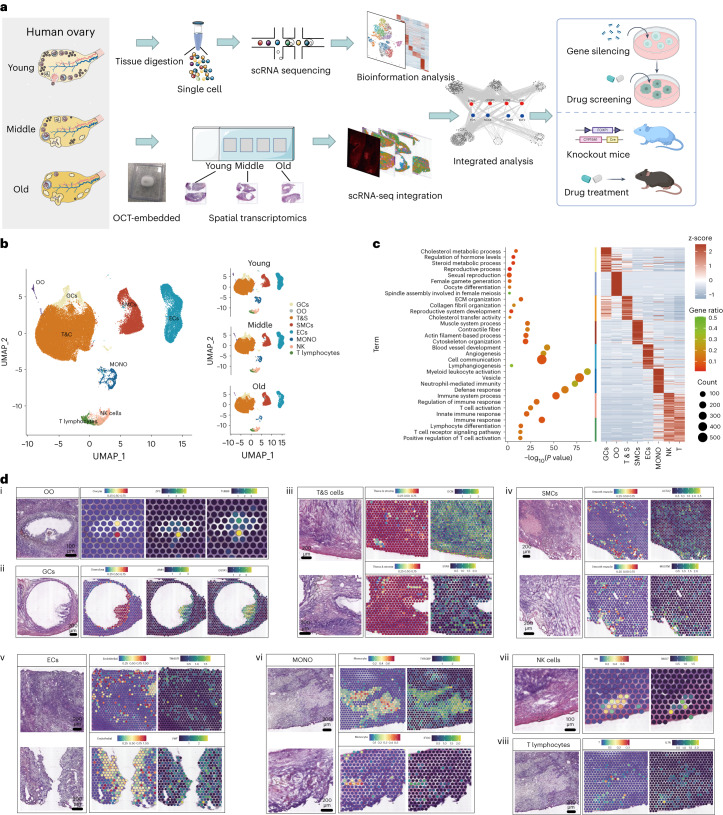
Single-cell transcriptome profiling and spatial location of human ovarian cells. **a**, The study flowchart. **b**, UMAP plots showing eight cell types (left) and age-dependent cell distribution (right). GC, granulosa cell; OO, oocyte; T&S, theca and stroma cells; SMCs, smooth muscle cells; EC, endothelial cell; MONO, monocytes. **c**, Representative GO terms of different cell types (left). Heat map showing top 50 marker genes in each cell type (right). *P* values were calculated by Fisher’s exact test. **d**, Each slide (i–viii) shows H&E staining, ST spot cell type predictions and characteristic markers, respectively of each cell type from left to right. The analysis was conducted in each ovarian tissue (*n* = 15).

**Extended Data Fig. 1:**
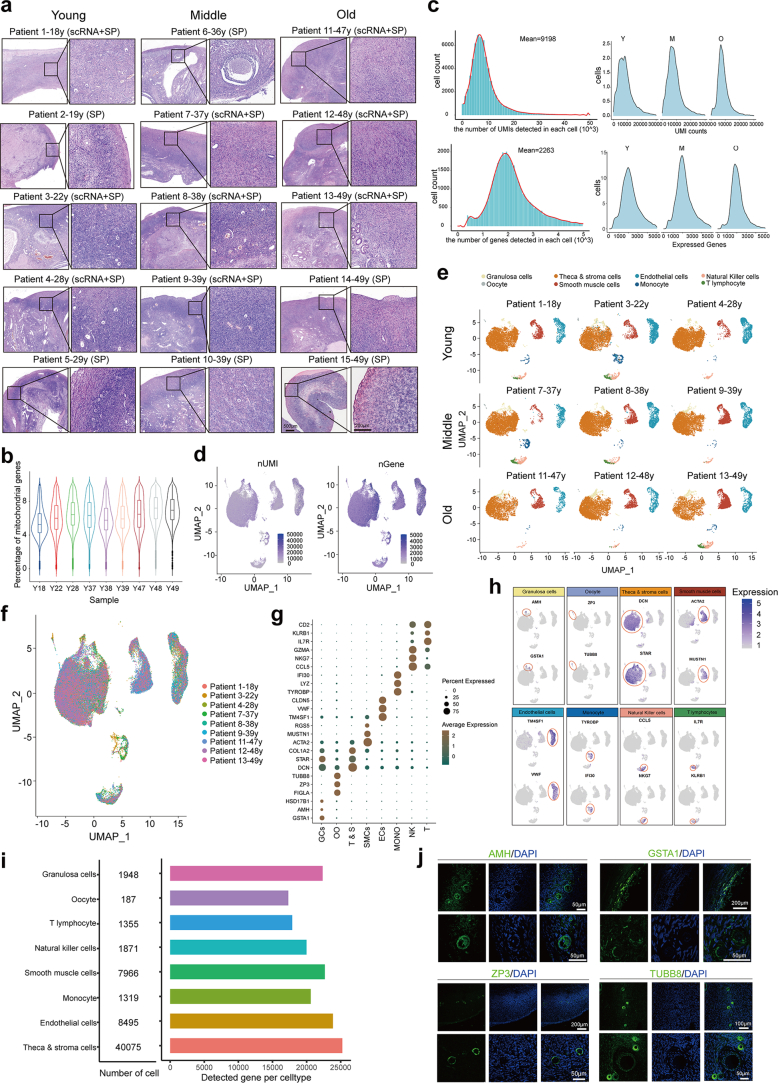
Information of human ovaries and quality control of scRNA-seq data. (**a**) H&E staining of ovaries from women in young, middle and old groups for scRNA-seq and ST-seq (n = 15). (**b**) Percentages of mitochondrial genes detected in each sample. Box-and-whisker plots (minimum, 25th percentile, median, 75th percentile, maximum). (**c**) Left: numbers of unique molecular identifiers (UMIs) (upper) and genes (lower) detected in each cell. Right: UMIs counts and expressed genes are shown by three groups. (**d**) The number of expressed UMIs (nUMI) and genes (nGene) in different cell types. (**e**) UMAP plot showing the eight ovarian cell clusters in each sample. (**f**) UMAP plot showing all ovarian cell clusters. (**g**) Top three markers of each cell type shown as expression quantity (circle size) and mean expression (color). (**h**) UMAP cluster map of each slide showing expression of marker genes. (**i**) Bar plot showing the cell numbers of different cell types and detected genes in human ovaries. (**j**) Immunofluorescence staining of human GCs markers (AMH and GSTA1) and oocytes markers (ZP3 and TUBB8). The experiment was repeated for three times.

The overview of the study design for the human ovarian aging atlas is shown in Fig. 1a. We collected a total of 15 healthy ovaries in the follicular phase from female volunteers who underwent hysterectomy and oophorectomy surgery due to cervical or endometrial cancer. Selected ovarian tissues were evaluated by two pathologists to exclude tumor metastasis (Extended Data Fig. 1a). The ovarian tissues of the 15 cases were divided into three groups based on age: young group (Y; 18–28 years), middle-aged group (M; 36–39 years) and older age group (O; 47–49 years). To further elucidate the dynamics of ovarian function, we assessed the level of serum anti-Müllerian hormone (AMH), a well-established clinical biomarker for evaluating ovarian reserve function. Notably, AMH levels were high in the young group (3.76 ng ml^−1^), declined in the middle-aged group (1.35 ng ml^−1^) and were nearly undetectable in the older age group (<0.06 ng ml^−1^) (Supplementary Table 1). We generated scRNA-seq data for nine ovarian tissues (from nine participants) and ST-seq data for 28 ovarian tissues (from 15 participants) (Supplementary Table 1).

### scRNA-seq profiling and spatial location of human ovarian cells

We used 10x Genomics to investigate ovarian aging at the single-cell level. Ovary tissues (0.5–1 cm³) from nine participants (three per age group) were enzymatically dissociated into individual cells, resulting in a total of 92,965 ovarian cells (31,005 from the young group, 32,557 from the middle-aged group and 29,403 from the older age group). Cells expressing high levels of mitochondrial genes (>10% of total unique molecular identifiers (UMIs)) were excluded (Extended Data Fig. 1b). The total cellular RNA content and number of expressed genes showed no significant difference among the three groups (Extended Data Fig. 1c). Then, we used the Uniform Manifold Approximation and Projection (UMAP) algorithm for the nonlinear dimensionality reduction analysis and identified eight cell types based on specific cell markers (Fig. 1b, Extended Data Fig. 1d–f and Supplementary Table 2). They were granulosa cells (GCs; *GSTA1*^*+*^, *AMH*^*+*^ and *HSD17B1*^*+*^), oocytes (*TUBB8*^*+*^, *ZP3*^*+*^ and *FIGLA*^*+*^) theca and stroma (T&S) cells (*DCN*^*+*^ and *STAR*^*+*^), smooth muscle cells (SMCs; *ACTA2*^*+*^ and *MUSTN1*^*+*^), endothelial cells (*TM4SF1*^*+*^ and *VWF*^*+*^), monocytes (*TYROBP*^*+*^ and *IFI30*^*+*^), natural killer (NK) cells (*CCL5*^*+*^ and *NKG7*^*+*^) and T lymphocytes (*IL7R*^*+*^ and *KLRB1*^*+*^) (Extended Data Fig. 1g,h). Similar to previous studies^6,7^, T&S cells accounted for the majority of ovarian cells (Extended Data Fig. 1i). We identified specific markers for oocytes and GCs, including the classical marker ZP3 and the oocyte-specific distribution of TUBB8, encoding a primary β-tubulin subunit. GSTA1, found specifically in GCs, may serve as a potential marker for GCs. To validate cell clustering accuracy, we conducted a Gene Ontology (GO) analysis of marker genes for each cell type (Fig. 1c). Notably, genes highly expressed in GCs were enriched in regulating hormone levels, whereas those in oocytes were linked to oocyte differentiation. T&S cells exhibited GO terms such as ‘extracellular matrix organization’ and ‘cholesterol transfer activity’ and SMCs showed enrichment in ‘muscle system process’ and ‘cytoskeleton organization’.

**Extended Data Fig. 2:**
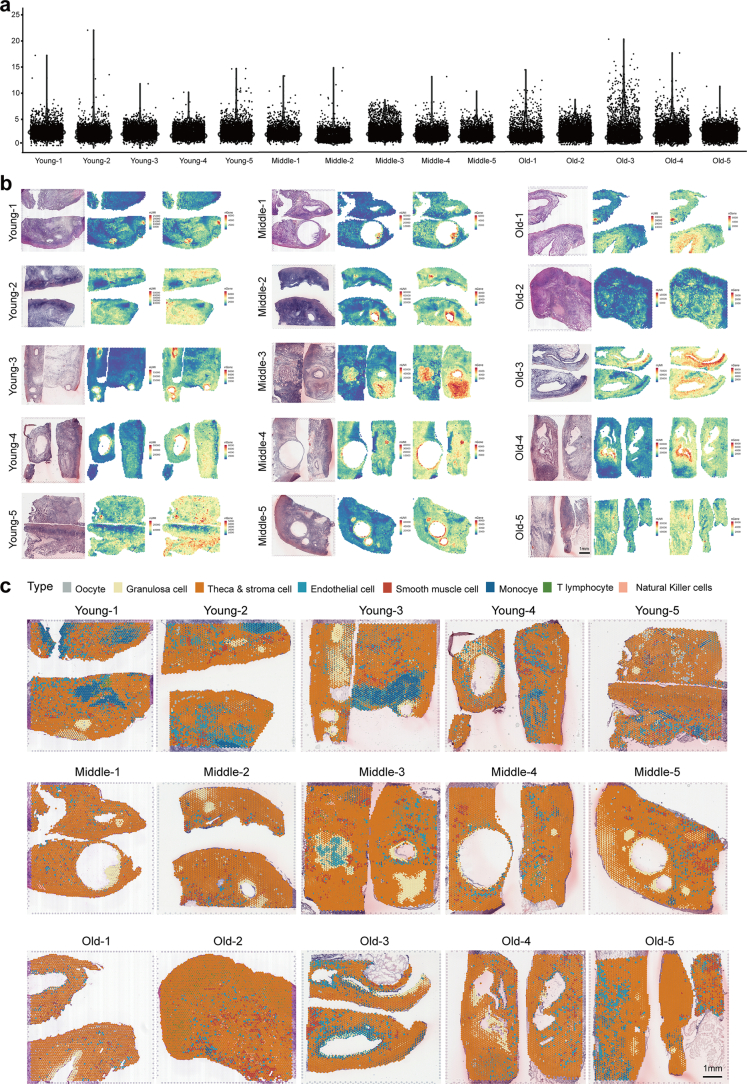
Quality control and summary of ST-seq data. (**a**) Violin plots showing the percentage of mitochondrial genes detected in each sample. (**b**) Spatial feature plots of the number of expressed UMI (nUMIs) and genes (nGene) in each sample. (**c**) Clustered ST spots integrated with scRNA-Seq cell type annotations mapped to the H&E image showing eight cell types in each sample.

Subsequently, ST sequencing (ST-seq) was conducted using ovarian tissues from 15 participants (five per group) to spatially map ovarian cell distributions. In the ST-seq data, the mtRNA of all spots represented less than 20% of total reads, indicating high data quality (Extended Data Fig. 2a). The ST-seq collectively identified over 8,000 genes (Extended Data Fig. 2b). Utilizing the scRNA-seq atlas, a factor analysis was performed to deduce the likely single-cell composition of each spot, effectively localizing all scRNA-seq clusters. Recognizable cell types, including oocytes centrally located in follicles (Fig. 1d(i)), GCs at the follicle periphery (Fig. 1d(ii)), widely distributed T&S cells (Fig. 1d(iii)) and SMC/endothelial cells along blood vessels (Fig. 1d(iv),(v)), were successfully mapped. The immune cells, including monocytes, NK cells and T lymphocytes, were mainly distributed in the interstitium of the medulla (Fig. 1d(vi),(vii),(viii)). The overall distribution of all ovarian cells was shown in Extended Data Fig. 2c. Spatial cell mapping revealed the spatial distribution of the clusters predicted by scRNA-seq of human ovary.

### Cell-specific changes in ovarian aging’s transcriptional programs

**Fig. 2:**
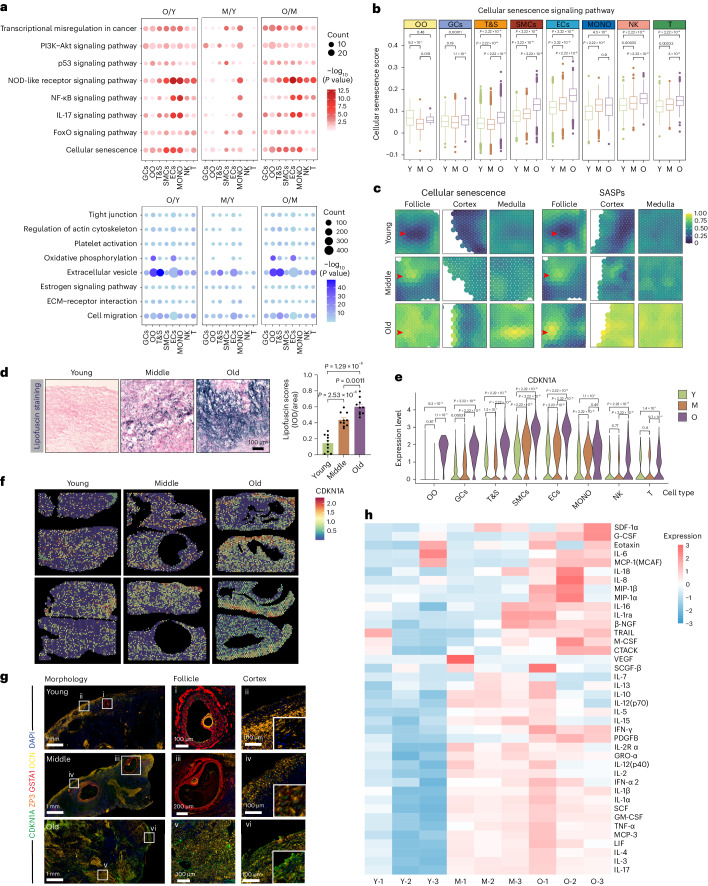
Cell type-specific spatiotemporal changes in transcriptional regulatory programs throughout ovarian aging. **a**, Representative KEGG pathways of upregulated DEGs (top) and downregulated DEGs (bottom) compared between the O/Y, M/Y and O/M groups in eight cell types. *P* values were calculated by Fisher’s exact test. **b**, Gene set score analysis of cellular senescence pathways in various ovarian cell types of different groups, Y, M and O. Data were analyzed by two-sided Wilcoxon rank-sum tests. Box-and-whisker plots show the minimum, 25th percentile, median, 75th percentile and maximum. *n* = 3 per age group. **c**, ST spot illustrating cellular senescence and SASP score within the human ovary. The red arrow indicates follicles. **d**, Lipofuscin staining of different aged human ovaries. IOD, integrated optical density. Data are presented as the mean ± s.e.m. *n* = 10 for each group (one-way ANOVA). **e**, Violin plot showing the expression of CDKN1A in all ovarian cell types. Data were analyzed by two-sided Wilcoxon rank-sum tests. **f**, ST spot indicating the CDKN1A expression in human ovaries of different ages. **g**, Multiplex IHC staining for CDKN1A, ZP3 (oocyte marker), GSTA1 (GC marker) and DCN (T&S cell marker) of differently aged human ovaries. The experiment was repeated three times. **h**, Cytokine oligonucleotide array for SASPs in human ovaries of different ages. *n* = 3 for each group. Source data

**Extended Data Fig. 3:**
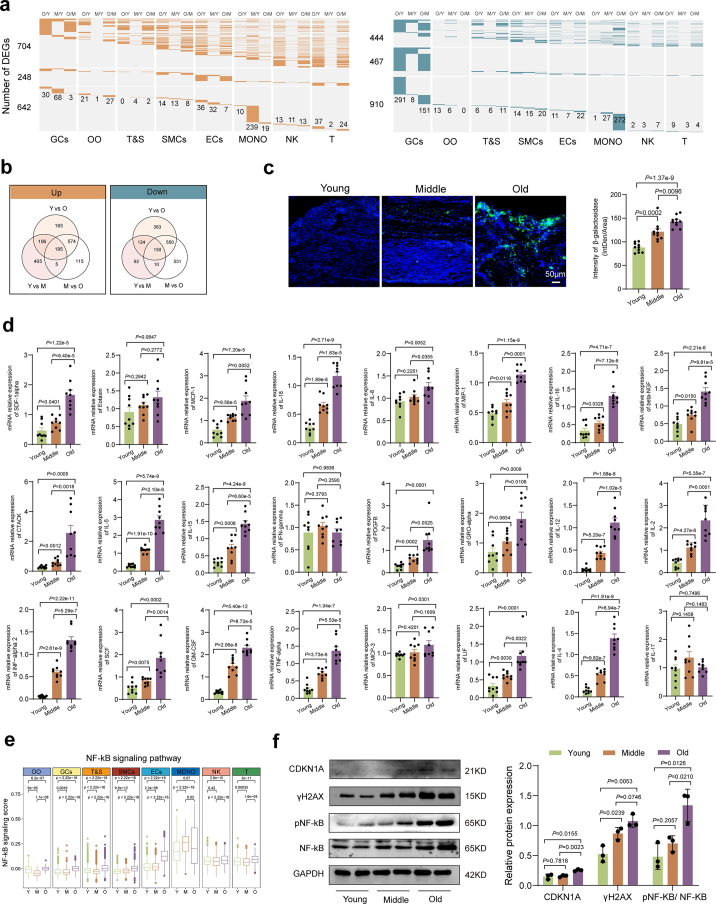
Supplement of gene expression in different cell types changed throughout human ovarian aging. (**a**) Heatmaps showing the upregulated (left) and downregulated (right) DEGs of each cell type between the old and young groups (O/Y), middle and young groups (M/Y), and old and middle groups (O/M). Gene numbers on the left represents from top to bottom, DEGs shared by at least two cell types, DEGs shared by at least two groups, unique DEGs of each cell type in each group. The numbers of unique DEGs are annotated. (**b**) Venn diagram of DEGs shared by three groups. Y, young; M, middle; O, old. (**c**) Fluorescence-based-β-Gal staining of human ovaries from young, middle and old group. The scores are listed on the right. Data are presented as the mean ± SEM. n = 10 for each group (one-way ANOVA). (**d**) Relative mRNA expression of SASPs in human ovaries at different ages by RT–qPCR. Data are presented as the mean ± SEM. n = 9 for each group (one-way ANOVA). (**e**) Gene set score analysis of NF-κB signaling pathway in eight ovarian cell types of different groups. Two-sided Wilcoxon rank-sum tests. Box-and-whisker plots (minimum, 25th percentile, median, 75th percentile, maximum). n = 3 per age group. (**f**) Protein expression of cellular senescence-related genes in human ovaries detected by Western blot. Representative images were shown. Data are presented as the mean ± SEM. This test was repeated three times (one-way ANOVA). Source data

We conducted a comparative analysis of gene expression patterns across various ovarian cell types in the young, middle-aged and older age groups. Differentially expressed genes (DEGs; |avg_logFC| > 0.25 and *P*_adj_ < 0.05) in at least one cell type were identified (Extended Data Fig. 3a and Supplementary Table 3). Specifically, a total of 1,068, 711 and 889 upregulated DEGs were identified between the young and old groups (O/Y), young and middle-aged groups (M/Y) and middle-aged and older age groups (O/M), respectively (Extended Data Fig. 3b). We also observed 1,187, 376 and 1,241 downregulated DEGs that were identified between the O/Y, M/Y and O/M groups, respectively (Extended Data Fig. 3b). Notably, the DEG analysis highlighted significant differences between perimenopausal ovaries and those with reproductive function in young or middle-aged stages. GO and KEGG enrichment analyses revealed that the upregulated DEGs were mainly associated with cellular senescence and some pathways associated with senescence, such as FoxO signaling pathway, IL-17 signaling pathway, nuclear factor (NF)-κB signaling pathway, NOD-like receptor signaling pathway, p53 signaling pathway and PI3K-Akt signaling pathway (Fig. 2a). The downregulated DEGs were mainly related to cell migration, extracellular matrix (ECM)–receptor interaction, estrogen signaling pathway, extracellular vesicle, oxidative phosphorylation, platelet activation, regulation of actin cytoskeleton and tight junction (Fig. 2a).

Based on the above results, cellular senescence may play an important role in human ovarian aging^18^. During the aging process, the signaling pathway score for senescence increased in the majority of ovarian cells (Fig. 2b). Pathway analysis of spatial gene expression data further indicated heightened enrichment of cellular senescence and a senescence-associated secretory phenotype (SASP) signaling pathway in the follicle, cortex and medulla of old ovaries (Fig. 2c). Notably, the accumulation of lipofuscin, a hallmark of cellular senescence, increased during ovarian aging. As shown in Fig. 2d, lipofuscin accumulation was increased during ovarian aging. Fluorescence-based β-galactosidase (β-gal) staining further illustrated a consistent increase throughout ovarian aging (Extended Data Fig. 3c). The major senescence hallmark CDKN1A/p21 showed increased expression in all ovarian cell types from the older age group (Fig. 2e). Spatial expression analysis using ST data confirmed the overall increase of CDKN1A during ovarian aging (Fig. 2f).

To pinpoint ovarian cell types expressing CDKN1A, multiplex immunohistochemistry (IHC) was employed. The findings revealed an accumulation of CDKN1A-positive cells during ovarian aging, notably in GCs and T&S cells, as evidenced by staining for GSTA1 and DCN (Fig. 2g). Examination of SASPs using cytokine oligonucleotide arrays revealed increased expression in aged ovaries, confirmed by quantitative PCR with reverse transcription (RT–qPCR) results. (Fig. 2h and Extended Data Fig. 3d). Activation of the NF-κB signaling pathway, known to induce SASPs and exacerbate cellular senescence, was observed in most ovarian cell types during aging (Extended Data Fig. 3e). Western blot results further supported the increase in markers related to cellular senescence (CDKN1A, γH2AX and pNF-κB) during ovarian aging (Extended Data Fig. 3f). In conclusion, these findings suggest that specific cell types in aged human ovaries may exhibit distinctive features associated with cellular senescence.

### Spatiotemporal changes in oocytes during ovarian aging

**Extended Data Fig. 4:**
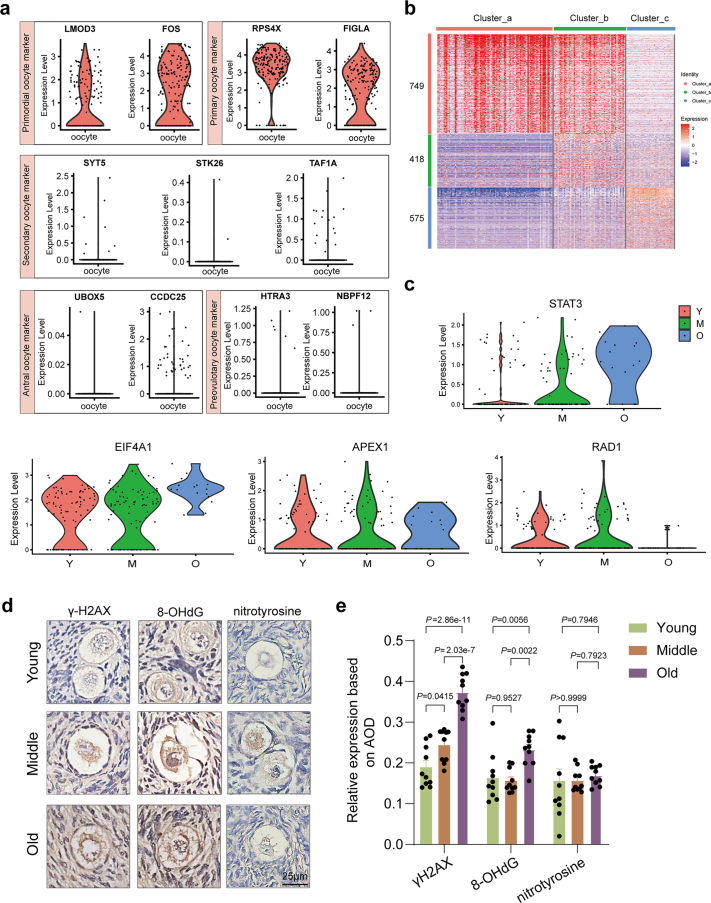
Supplement of gene expression in oocytes changed throughout human ovarian aging. (**a**) The expression level of stage-specific markers for oocytes. LMOD3 and FOS for primordial oocytes, RPS4X and FIGLA for primary oocytes, SYT5, STK26 and TAF1A for secondary oocytes, UBOX5 and CCDC25 for antral oocytes, HTRA3 and NBPF12 for preovulotary oocytes. (**b**) Heat map illustrating the DEGs of each oocyte stage. DEG numbers are shown on the left. (**c**) Representative genes of DNA damage (STAT3, EIF4A1) and DNA repair (APEX1, RAD1) expression in oocytes of three groups. Y, young; M, middle; O, old. (**d**) Representative images of oocytes by IHC of γH2AX, 8-OHdG and nitrotyrosine between three groups.(**e**) IHC scores of relative expression for each group. AOD, average optical density. Data are presented as the mean ± SEM. n = 10 for each group (one-way ANOVA).

Due to microfluidic channel limitations on the 10x Genomics platform, only oocytes with a diameter less than 50 µm could be obtained. To determine the oocyte stage, previously reported stage-specific markers, including LMOD3 and FOS for primordial oocytes, RPS4X and FIGLA for primary oocytes, SYT5, STK26 and TAF1A for secondary oocytes, UBOX5 and CCDC25 for antral oocytes, and HTRA3 and NBPF12 for preovulatory oocytes, were analyzed^8,19^. scRNA-seq results indicated high expression of LMOD3, FOS, RPS4X and FIGLA in these oocytes, suggesting that they were at the primordial or primary stage (Extended Data Fig. 4a).

**Fig. 3:**
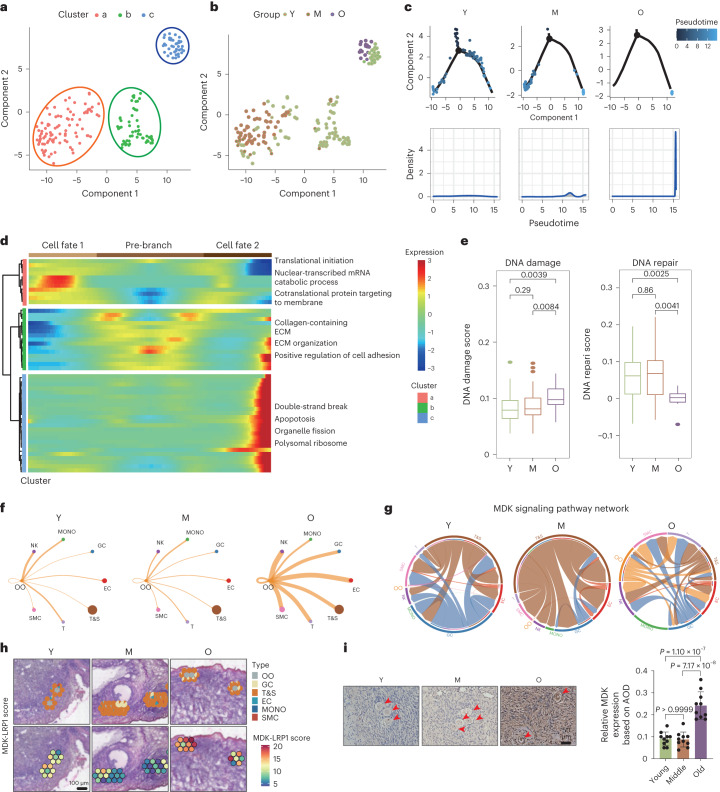
Spatiotemporal changes of oocytes during aging. **a**, Pseudotime trajectory plot of oocytes. **b**, UMAP plot of oocytes from young, middle-aged and older age groups. **c**, Two-dimensional (2D) graph of the pseudotime-ordered oocyte cells from young, middle-aged and older age groups. **d**, Heat map showing the dynamic DEGs along the pseudotime. The related biological process of each subtype is listed on the right. **e**, Gene set score analysis of DNA damage and DNA repair pathways in oocytes of different groups. Data were analyzed by two-sided Wilcoxon rank-sum tests. Box-and-whisker plots show the minimum, 25th percentile, median, 75th percentile and maximum. *n* = 3 per age group. **f**, Cell communication analysis of oocytes between other ovarian cells. **g**, MDK signaling pathway network of oocytes between other ovarian cells in Y, M and O groups. **h**, ST spot showing the score of MDK-LRP1 in oocytes and their surround cells in ovaries of three groups, Y, M and O. The analysis was conducted in each ovarian tissue (*n* = 15). **i**, Representative image of oocytes from Y, M and O human ovary sections stained for MDK protein by IHC. The red arrows indicate primordial oocytes. The scores are listed on the right. Data are presented as the mean ± s.e.m. *n* = 10 for each group (one-way ANOVA). Source data

Pseudotime trajectories were analyzed for oocytes, identifying three stages (cluster a, b and c) (Fig. 3a). Oocytes in the young group exhibited distribution across the three types, whereas those in the middle-aged group were primarily in cluster a, and all oocytes in the older age group were in cluster c (Fig. 3b). Pseudotime analysis revealed that oocytes in the young group were at the trajectory’s beginning, whereas those in the old group were in a terminal state, suggesting GeneSwitch during aging (Fig. 3c). Dynamic changes in gene expression patterns at each stage were analyzed, revealing 749, 418 and 575 DEGs (*P*_adj_ < 0.05, log_2_FC > 0.5) in the three stages, respectively (Extended Data Fig. 4b). In cluster a, enriched GO terms included ‘cotranslational protein targeting to membrane,’ ‘nuclear-transcribed mRNA catabolic process’ and ‘translational initiation’ (Fig. 3d). Cluster b showed enrichment in ‘collagen-containing extracellular matrix,’ ‘extracellular matrix organization’ and ‘positive regulation of cell adhesion’ (Fig. 3d). In cluster c, enriched GO terms included ‘apoptosis,’ ‘double-strand break repair’ and ‘organelle fission,’ indicating DNA damage and apoptosis in oocytes (Fig. 3d).

Next, we compared the DNA damage and repair gene list and generated scores during oocyte aging. DNA damage scores increased and DNA repair scores decreased in aged oocytes (Fig. 3e). Expression of classical DNA damage response genes (for example, STAT3 and EIF4A1) significantly increased in older oocytes, whereas crucial DNA repair genes (for example, APEX1 and RAD1) were downregulated (Extended Data Fig. 4c). IHC results supported DNA oxidation (8-OHdG-positive) and DNA damage (γH2AX-positive) accumulation in aged oocytes at the primordial stage (Extended Data Fig. 4d,e). Overall, increased DNA damage and diminished DNA repair may contribute to aging in primordial or primary stage oocytes.

**Extended Data Fig. 5:**
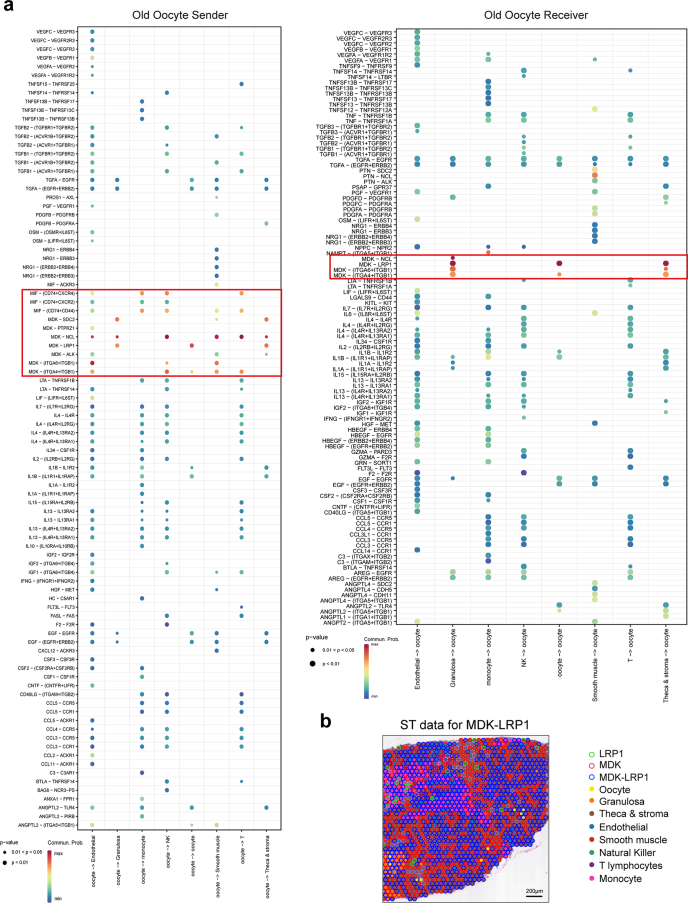
Supplement of cell communications in oocytes. (**a**) The receptor-ligands of communication between oocytes and other ovarian cells in the old group. P values were calculated by Fisher’s exact test. (**b**) ST spot showing the expression of receptor–ligand pair MDK and LRP1 in human ovary.

Subsequently, CellPhoneDB was employed to investigate cell communication between oocytes and other ovarian cell types during aging. Notably, interactions between oocytes and other cell types were significantly enhanced in aged groups (Fig. 3f). Aged oocytes exhibited elevated expression of the receptor–ligand pair MDK-LRP1, known for its crucial role in chronic inflammation and the recruitment of inflammatory cells^20^ (Fig. 3g and Extended Data Fig. 5a). Further exploration of cell communication, focusing on the coexpression of MDK and LRP1 within ST spots, revealed widespread expression in both oocytes and surrounding cells, with higher scores observed in the older age groups (Extended Data Fig. 5b and Fig. 3h), consistent with IHC staining results (Fig. 3i). These findings suggest a potential association between increased MDK-LRP1 expression and heightened cell communication between oocytes and other ovarian cells in aged human ovaries. The interactions identified in our data may contribute to an inflammatory response, thereby exacerbating DNA damage in oocytes during aging.

### Spatiotemporal transcriptional changes of GCs during aging

**Fig. 4:**
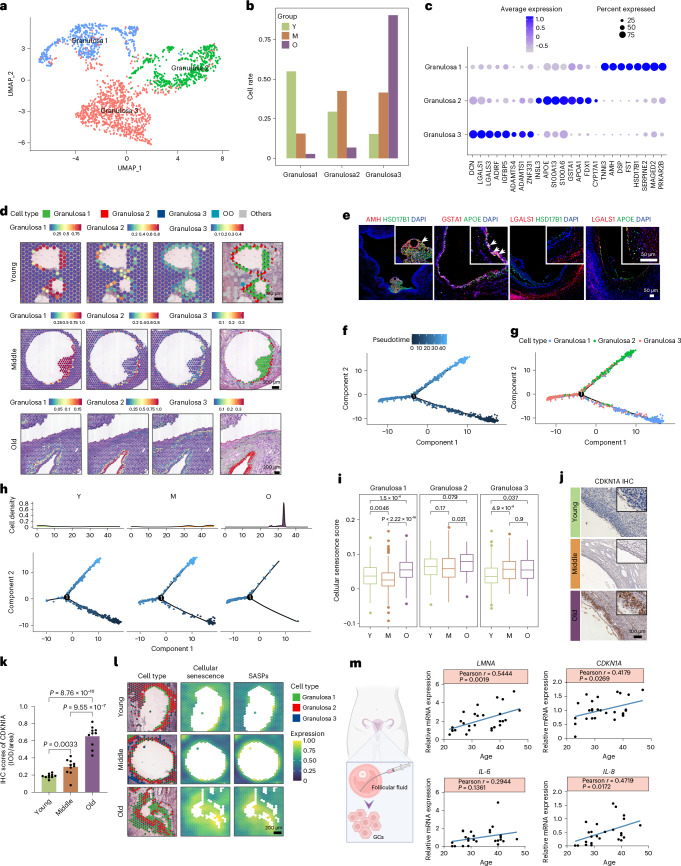
Spatiotemporal transcriptional changes of three GCs subpopulations during aging. **a**, UMAP visualization of GC subclusters. **b**, Histogram showing the cell rate of three GC subclusters in Y, M and O groups. **c**, Dot plot heat map showing top eight markers for subcluster GCs. **d**, The spatial cluster distribution of each subclusters (left) and ST spot overlapped with H&E staining (right). **e**, Multiplex IHC staining for the markers of subtype GCs. AMH (granulosa 1), GSTA1 (granulosa 2), LGALS1 (granulosa 3). The experiment was repeated three times. **f**,**g**, Pseudotime-ordered analysis of GCs. **h**, Two-dimensional graph of the pseudotime-ordered GCs from Y, M and O groups. The cell density distribution is shown in the above figure. **i**, Gene set score analysis of cellular senescence pathways in GC subtypes with age. Data were analyzed by two-sided Wilcoxon rank-sum tests. Box-and-whisker plots show the minimum, 25th percentile, median, 75th percentile and maximum. *n* = 3 per age group. **j**, Representative images of CDKN1A expression in GCs of three groups by IHC. **k**, IHC scores for CDKN1A in GCs. Data are presented as the mean ± s.e.m. (one-way ANOVA). *n* = 10 for each group. **l**, ST spot overlay of cellular senescence and SASP gene set score in GCs of three groups. **m**, The correlation analysis of SASP levels with age in primary human GCs (hGCs). *n* = 25–28 (Pearson correlation analysis, two-sided). Source data

**Extended Data Fig. 6:**
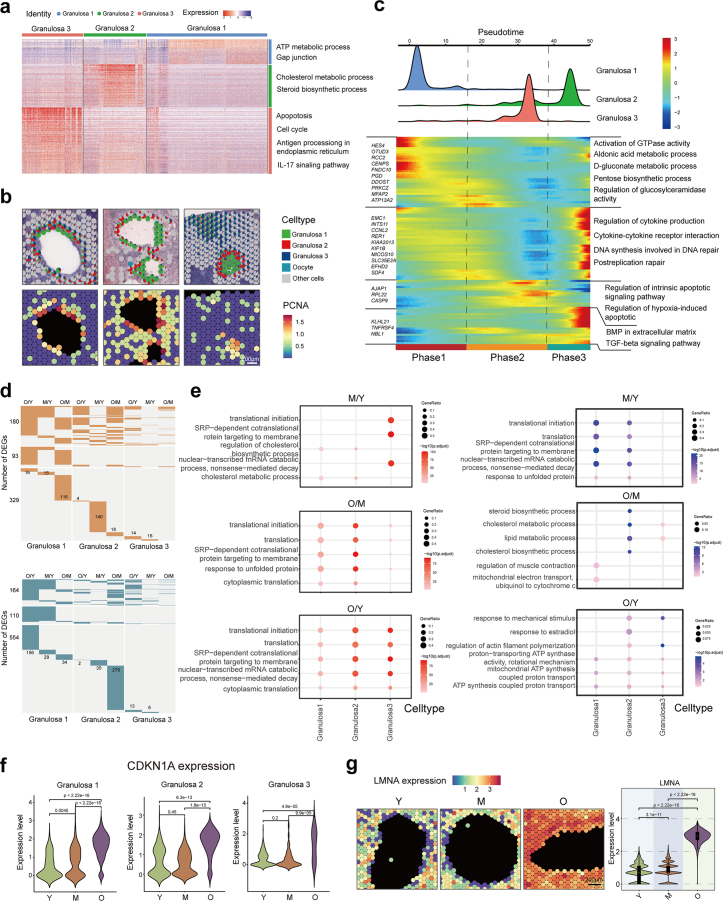
Supplement of GCs subclusters. (**a**) Heat map showing the highly expressed genes specifically in granulosa 1, granulosa 2, granulosa 3. (**b**) ST spot overlay of PCNA expression in GCs subtypes. (**c**) Heat map showing the dynamic DEGs along the pseudotime. Subtypes are labeled by colors (upper panel). The related biological process of each subtype is listed on the right. The related genes are listed on the left. (**d**) Heatmaps showing the upregulated (above, orange) and downregulated (below, blue) DEGs for each GCs subclusters between three groups (O/Y, M/Y, O/M). (**e**) Representative GO terms of DEGs enrichment between three groups (O/Y, M/Y, O/M) in each GCs subtypes. Left, the GO terms of upregulated DEGs. Right, the GO terms of downregulated DEGs. P values were calculated by Fisher’s exact test. (**f**) Violin plot showing the expression of CDKN1A in three GCs subclusters during aging. Two-sided Wilcoxon rank-sum tests. (**g**) ST spot overlay of LMNA expression in GCs of three groups. The ST data analysis is shown on the right. Two-sided Wilcoxon rank-sum tests. Box-and-whisker plots (minimum, 25th percentile, median, 75th percentile, maximum). n = 5 per age group.

GCs, which envelop and interact with the developing oocyte, have unclear classifications and changes during aging. UMAP analysis categorized all GCs into three subtypes (GC subtypes 1, 2 and 3) (Fig. 4a and Supplementary Table 2). Analysis indicated that GCs in the young group were predominantly GC subtypes 1 and 2, whereas those in the older age group were mainly GC subtype 3 (Fig. 4b). Further analysis revealed that GC subtype 1 expressed known markers such as *AMH*, *FST*, *HSD17B1*, *SERPINE2* and *PRKAR2B*, along with previously unreported genes such as DSP and MAGED2 (Fig. 4c). GO analysis highlighted enrichment in ‘ATP metabolic process’ and ‘gap junction’, processes involved in follicular development, in GC subtype 1 (Extended Data Fig. 6a). GC subtype 2 exhibited expression of genes related to hormone synthesis, including *INSL3*, *APOE*, *GSTA1*, *APOA1*, *FDX1* and *CYP17A1* (Fig. 4c). GO terms such as ‘cholesterol metabolic process’ and ‘steroid biosynthetic process’ were enriched in subtype 2 (Extended Data Fig. 6a). GC subtype 3 expressed markers *DCN*, *LGALS1* and *LGALS3*, which are known regulators of GC apoptosis and the cell cycle^21,22^ (Fig. 4c). GO terms ‘apoptosis’ and ‘cell cycle’ were enriched in subtype 3 (Extended Data Fig. 6a). These findings provide insights into the transcriptional characteristics of three subpopulations of GCs in the human ovary.

Next, we transferred labels from the integrated data to spatial gene expression data, mapping GC subtypes based on spatial location. Notably, the ST data revealed three distinct populations of GCs in different areas. GC subtype 1 was situated in the cumulus of the antral follicle, subtype 2 was in in the mural layer of follicles and subtype 3 exhibited a widespread distribution across antral follicles (Fig. 4d). Experimental validation using subtype-specific markers (AMH for subtype 1, GSTA1 for subtype 2 and LGALS1 for subtype 3) confirmed these distinct spatial distributions (Fig. 4e). Within GC subtype 1, the expression of the steroidogenic enzyme gene HSD17B1, crucial for steroidogenesis in GCs, was notably high (Fig. 4e). Subtype 2 expressed APOE, a key player in cholesterol transport during steroidogenesis^23^, at high levels (Fig. 4e). Neither HSD17B1 nor APOE was expressed in GC subtype 3, indicating reduced hormone synthesis in this subtype. The ST data also revealed that PCNA, a marker of cell proliferation, was highly expressed in GC subtypes 1 and 2, but was low in subtype 3, suggesting that subtypes 1 and 2 have a stronger proliferative ability than subtype 3 (Extended Data Fig. 6b).

Subsequently, we explored dynamic states and cell transitions in GCs by inferring state trajectories using Monocle. The analysis revealed that GC subtype 1 was at the beginning of the trajectory path (Fig. 4f,g). The early-stage GC subtype 1 was predominantly distributed in young and middle-aged ovaries, whereas GCs in old-aged samples were primarily at the terminal ends of GC subtype 3, indicating GeneSwitch in GCs during ovarian aging (Fig. 4h). Transcriptional changes associated with transitional states were investigated, categorizing GC clusters into three phases (Extended Data Fig. 6c). Subtype 1 was predominantly in phase 1, with signaling pathways related to the metabolism of guanosine triphosphate (GTP) and glucose. Subtype 2 was mainly in phase 3, characterized by genes involved in cytokine production and the ECM. Subtype 3 was mainly in phase 2, enriched in the apoptosis pathway. Overall, these results suggested that GC subtypes 1 and 2 were predominantly observed in young and middle-aged ovaries, whereas subtype 3 was more prevalent in older ovaries. This shift in composition may imply a decrease in functional GC types and a potential increase in apoptotic GCs in aging ovaries.

In examining aging-related gene expression changes in GCs, further analysis of transcriptomic alterations in these subpopulations revealed partially overlapping DEGs in GC subtypes between the young, middle-aged and older age groups (Extended Data Fig. 6d). From young to middle age, downregulated DEGs in GC subtypes 1 and 2 were associated with transcriptional and translational signaling, whereas they were upregulated in GC subtype 3 (Extended Data Fig. 6e). From middle to old age, upregulated DEGs in GC subtypes 1 and 2 were associated with the aforementioned transcriptional and translational signaling. The downregulated DEGs in GC subtype 2 were enriched in processes associated with steroidogenesis, including steroid biosynthetic process, cholesterol metabolic process, lipid metabolic process and cholesterol biosynthetic process.

To investigate whether cellular senescence undergoes changes in GC subtypes during aging, we computed the cellular senescence score, revealing an augmentation in all three types of GCs in the aged ovary (Fig. 4i). Correspondingly, CDKN1A was upregulated in all three subtypes of GCs (Extended Data Fig. 6f). Immunostaining analyses confirmed increased levels of CDKN1A protein in aged ovarian GCs (Fig. 4j). Then, we assessed cellular senescence and SASP scores of GCs in our ST data. GCs in the older age group exhibited higher levels of cellular senescence and SASPs compared to the young and middle-aged groups, especially in GC subtype 1 (Fig. 4k). Based on the analysis of ST data, LMNA may be an important marker for GC senescence (Extended Data Fig. 6g). Additionally, primary human GCs (hGCs) were obtained from participants aged 21 to 47 years old who underwent assisted reproductive therapy to further explore cellular senescence (Fig. 4l). SASP expression in hGCs increased progressively with ages ranging from 22 to 47 years (LMNA, *P* = 0.0019; CDKN1A, *P* = 0.0269; IL-8, *P* = 0.0172). These findings suggest that cellular senescence is a potential feature of aging GCs, which may contribute to ovarian dysfunction during aging.

### Spatiotemporal changes of T&S cells during aging

**Fig. 5:**
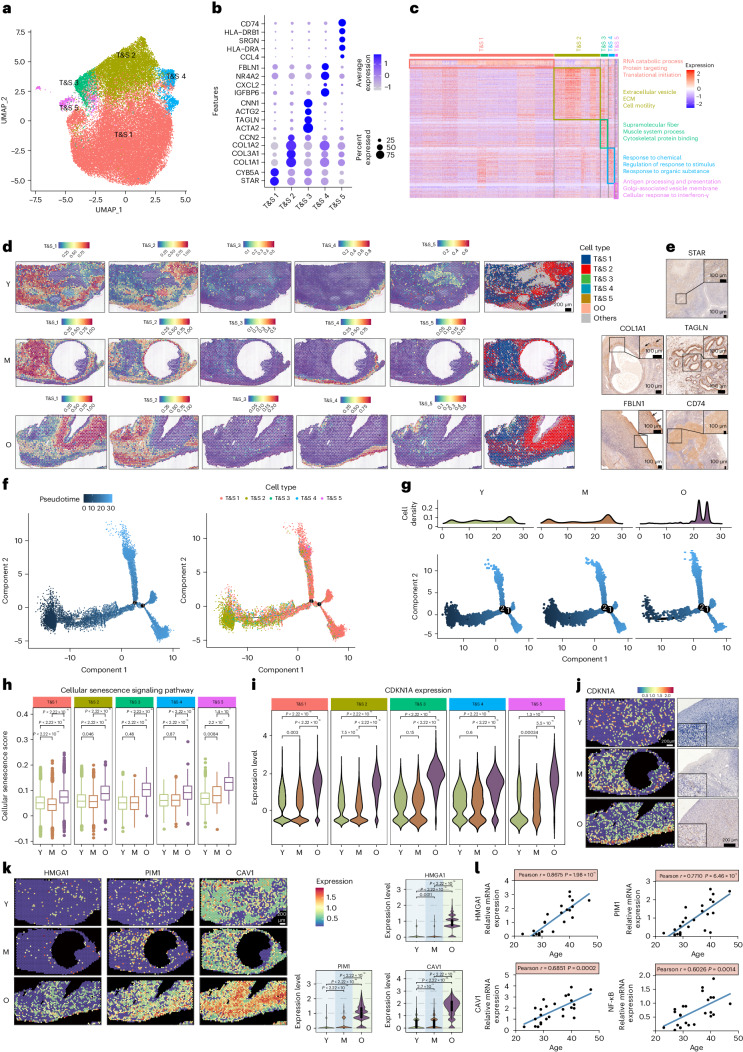
Spatiotemporal changes of five T&S cells subpopulations during aging. **a**, UMAP visualization of T&S cell subclusters. **b**, Dot plot heat map showing top T&S cell subclusters markers. **c**, Heat map showing the highly expressed genes and GO terms specifically in T&S subtype cells. **d**, The spatial cluster distribution of each subclusters (left) and ST spot overlapped with H&E staining (right). **e**, IHC for markers of T&S subtype cells. The experiment was repeated three times. **f**, Pseudotime-ordered analysis of T&S cells (left). Subtypes are labeled by colors (right). **g**, A 2D graph of the pseudotime-ordered T&S cells from the young, middle and older age groups. The cell density distribution is shown in the above figure. **h**, Gene set score analysis of cellular senescence pathways in T&S subtype cells with age. Data were analyzed by two-sided Wilcoxon rank-sum tests. Box-and-whisker plots show the minimum, 25th percentile, median, 75th percentile and maximum. *n* = 3 per age group. **i**, Violin plot showing the expression of CDKN1A in five T&S cells subclusters. Data were analyzed by two-sided Wilcoxon rank-sum tests. **j**, ST spot overlay of CDKN1A expression (left) and representative images of CDKN1A expression by IHC (right) in T&S cells of three groups. **k**, ST spot overlay of SASPs (HMGA1, PIM1 and CAV1) expression in T&S cells of three groups. The ST data analysis is shown on the right. Data were analyzed by two-sided Wilcoxon rank-sum tests. The analysis was conducted in each ovarian tissue (*n* = 15). **l**, The correlation analysis of SASPs levels with age in pT&S cells from human ovaries*. n* = 25 (Pearson correlation analysis, two-sided). Source data

**Extended Data Fig. 7:**
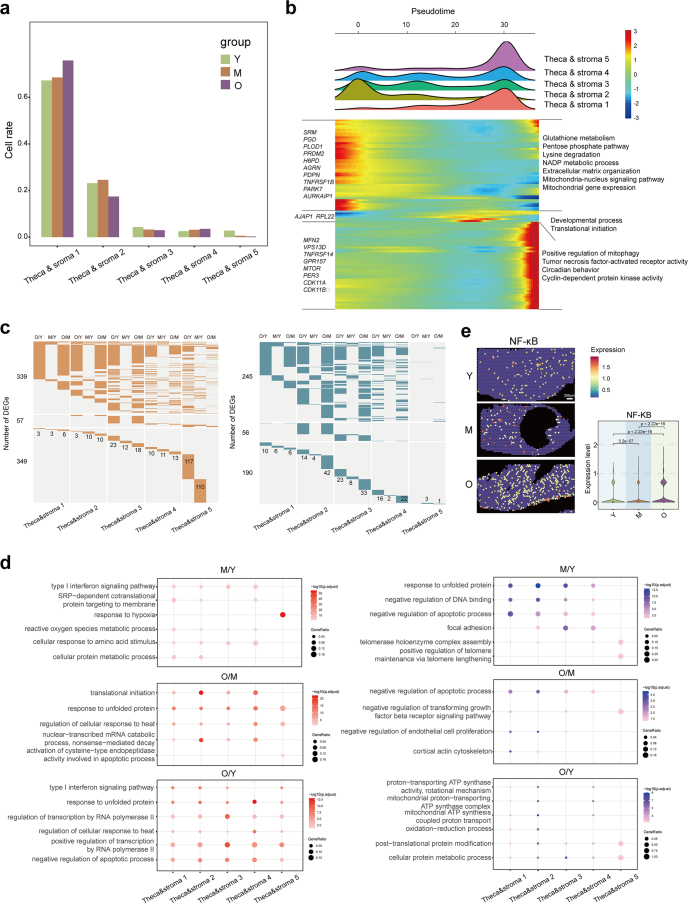
Supplement of T&S cells subclusters. (**a**) Histogram showing the cell rate of five T&S subclusters in young (Y), middle (M) and old (O) groups. (**b**) Heat map showing the dynamic DEGs along the pseudotime. Subtypes are labeled by colors (upper panel). The related biological process of each subtype is listed on the right. The related genes are listed on the left. (**c**) Heatmaps showing the upregulated (left, orange) and downregulated (right, blue) DEGs for each T&S subclusters between three groups (O/Y, M/Y, O/M). (**d**) Representative GO terms of DEGs enrichment between three groups (O/Y, M/Y, O/M) in each theca & stroma cell subtype. Left, the GO terms of upregulated DEGs. Right, the GO terms of downregulated DEGs. P values were calculated by Fisher’s exact test. (**e**) ST data analysis for NF-κB in T&S cells of three groups. Y, young; M, middle; O, old. Two-sided Wilcoxon rank-sum tests.

The ovary contains a significant stromal compartment (Extended Data Fig. 2c), with unknown classification and aging-related changes in ovarian stromal cells. Through UMAP analyses, we identified five T&S cell subtypes with distinct transcriptomic signatures (Fig. 5a and Supplementary Table 2). Subtype 1, characterized by *STAR* and *CYB5A* expression, exhibited enriched RNA and protein synthesis processes (Fig. 5b,c) and widespread distribution in the ovarian medulla (Fig. 5d). Subtype 2, expressing ECM genes such as *COL1A1* and *COL3A1*, was associated with ‘extracellular vesicle’ and ‘extracellular matrix’ pathways and located around follicles (Fig. 5b–d). Subtype 3, expressing myofibroblast markers *ACTA2* and *TAGLN*, showed enrichment in supramolecular fiber pathways and cytoskeletal protein binding, located around blood vessels (Fig. 5b–d). Subtype 4, defined by *FBLN1* and *CXCL2* expression, was associated with ‘response to chemical’ and ‘regulation of response to stimulus’ pathways, located in the outer cortex, potentially involved in ovarian defense against external stress (Fig. 5b–d). Subtype 5, characterized as inflammatory-like fibroblasts expressing *CD74*, *HLA-DRB1*, *CCL4* and *HLA-DRA*, was enriched in pathways related to ‘antigen processing and presentation’, ‘cellular response to interferon-γ’ and complement cascades, may be involved in post-ovulation repair. Further experimental verification was conducted by using the markers of T&S subtype cells (Fig. 5e). Statistics indicate decreased numbers of T&S cells subtype 2 and 5 in the older age group, aligning with reduced follicles and ovulation in aged ovaries (Extended Data Fig. 7a). Overall, these findings suggest the existence of five distinct T&S cell clusters, with their spatiotemporal dynamics likely linked to their functions.

We investigated the dynamic states and cell transitions in T&S cells by analyzing state trajectories. Subtype 2 of T&S cells occupied the initial phase of the trajectory path (Fig. 5f). The clusters of T&S cells were divided into three phases based on transcriptional changes, with subtype 2 mainly representing phase 1 cells involved in metabolic processes and the ECM, aligning with their role in follicle development (Extended Data Fig. 7b). Additionally, we analyzed the trajectories of T&S cells in young, middle-aged and older age groups. Notably, early-stage T&S cells were predominantly present in young and middle-aged samples, whereas those in old-aged samples were mainly found at the terminal ends of the transition path, suggesting potential changes in the types of T&S cells during the aging process (Fig. 5g).

Additionally, we investigated age-related changes in gene expression within the T&S cells. Examination of transcriptomic changes during aging revealed partially overlapping DEGs among subpopulations (Extended Data Fig. 7c). Subtypes 1–4 of T&S cells exhibited similar gene expression changes from young to middle age, with upregulated DEGs linked to pro-inflammatory pathways (Extended Data Fig. 7d). In subtype 5, upregulated DEGs were associated with hypoxia response, whereas downregulated DEGs were involved in telomere function. Transitioning from middle to old age, all T&S cells subtypes displayed upregulated DEGs enriched in cellular stress response, and subtypes 1–4 had downregulated DEGs associated with the negative regulation of apoptosis. Subtype 5 exhibited downregulated DEGs related to fibrotic signaling. These findings underscore the emergence of transcriptional signatures associated with inflammation, apoptosis and fibrosis in aged T&S cells.

The cellular senescence score increased across all T&S cell subpopulations during aging (Fig. 5h), accompanied by upregulated CDKN1A expression in aged cells (Fig. 5i). ST data confirmed elevated CDKN1A, especially in subtype 4 located in the cortex, aligning with IHC results (Fig. 5j, left and right). Analyzing ST data further revealed a significant rise in SASP factors, including HMGA1, PIM1 and CAV1, in aged T&S cells (Fig. 5k). NF-κB signaling, known to stimulate SASPs, was also increased in the T&S cells of the older age group (Extended Data Fig. 7e). To validate SASP upregulation in T&S cells during aging, primary T&S (pT&S) cells from ovaries of women aged 23 to 47 years exhibited aging-associated increases in HMGA1, PIM1, CAV1 and NF-κB, consistent with scRNA-seq and ST analysis (Fig. 5l). These findings collectively suggest the presence of senescence-like characteristics in T&S cells during ovarian aging.

### FOXP1 is a core transcriptional regulator for ovarian cellular senescence in vitro

**Fig. 6:**
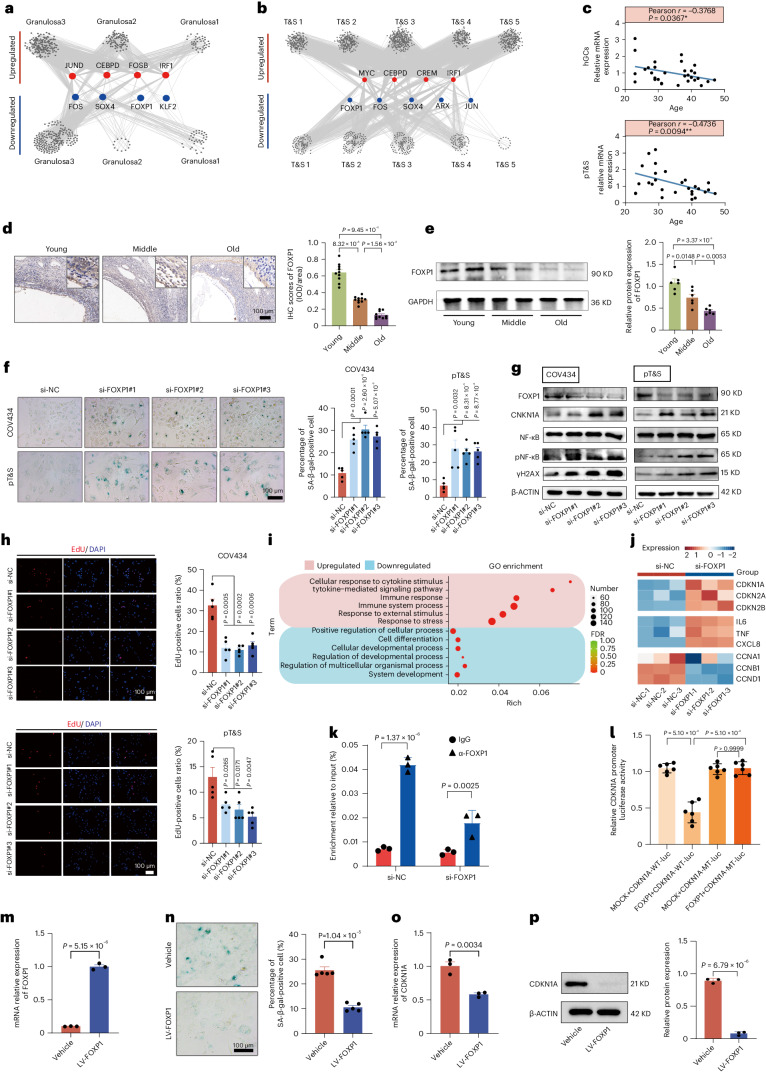
The role of FOXP1 in ovarian aging. **a**, Network of regulatory TFs in three GC subtypes. Node size correlates with the number of edges positively. **b**, Network of regulatory TFs in five T&S subtypes. **c**, The correlation analysis of FOXP1 expression with age in hGCs and pT&S. *n* = 28–30 (Pearson correlation analysis, two-sided). **d**, FOXP1 expression in ovaries. Data are presented as the mean ± s.e.m. *n* = 10 for each group (one-way ANOVA). **e**, Western blotting of FOXP1 protein levels in ovaries. Data are presented as the mean ± s.e.m. *n* = 6 for each group (one-way ANOVA). **f**, SA-β-gal staining. Data are presented as the mean ± s.e.m. *n* = 5 for each group (unpaired two-tailed *t*-test). **g**, Protein expression of genes related to cellular senescence. The experiment was repeated for three times. **h**, EdU incorporation assay. Data are presented as the mean ± s.e.m. *n* = 5 for each group (unpaired two-tailed *t*-test). **i**, Representative GO terms of DEGs. *P* values were calculated by Fisher’s exact test. **j**, The expression of SASPs and cell cycle-related genes. **k**, ChIP–qPCR using the FOXP1 antibody at the CDKN1A promoter. Data are presented as the mean ± s.e.m. *n* = 3 for each group (unpaired two-tailed *t*-test). **l**, Activity of WT and mutant (MT) CDKN1A promoter luciferase (luc) reporter. Empty vector-infected cells (MOCK) served as the control. Data are presented as the mean ± s.e.m. *n* = 6 for each group (one-way ANOVA). **m**, mRNA expression after FOXP1 overexpression in COV434. Data are presented as the mean ± s.e.m. *n* = 3 for each group (unpaired two-tailed *t*-test). **n**, SA-β-gal staining. The percentage of SA-β-gal-positive cells was shown on the right. Data are presented as the mean ± s.e.m. *n* = 5 for each group (unpaired two-tailed *t*-test). **o**, mRNA expression of CDKN1A. Data are presented as the mean ± s.e.m. *n* = 3 for each group (unpaired two-tailed *t*-test). **p**, The protein expression of CDKN1A in COV434. Data are presented as the mean ± s.e.m. This test was repeated three times (unpaired two-tailed *t*-test). Source data

**Extended Data Fig. 8:**
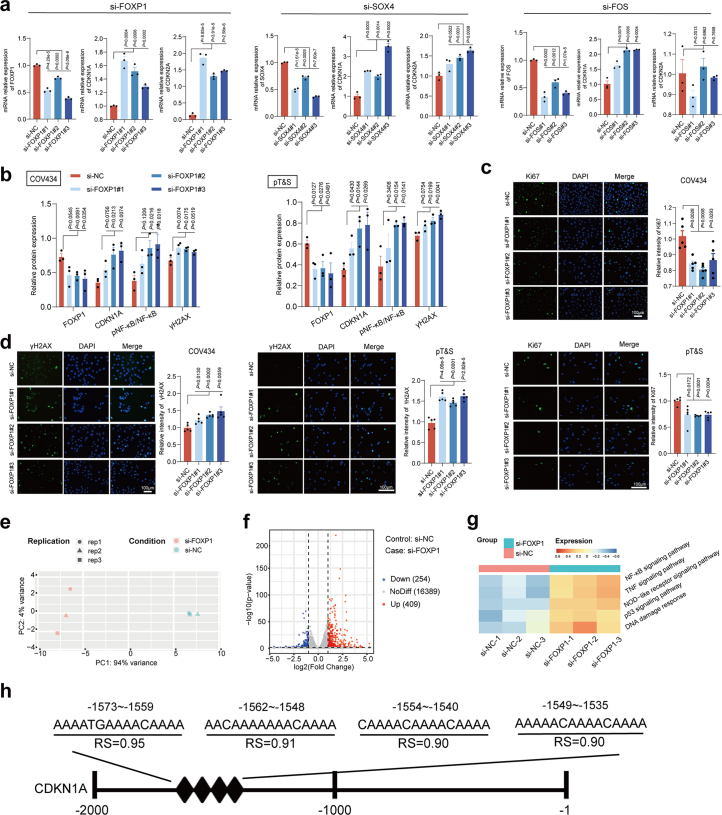
Supplement of transcription factor. (**a**) Relative mRNA expression of FOXP1, SOX4, FOS-knockdown COV434 cells. Data are presented as the mean ± SEM. n = 3 for each group (unpaired two-tailed t-test). (**b**) Analysis of protein expression of genes related to cellular senescence in COV434 and pT&S upon si-FOXP1-mediated gene knockdown. Data are presented as the mean ± SEM. n = 3 for each group (unpaired two-tailed t-test). (**c**) Immunofluorescence staining of Ki67 in COV434 and pT&S upon si-FOXP1-mediated gene knockdown. The relative intensity is listed on the right. Data are presented as the mean ± SEM. n = 5 for each group (unpaired two-tailed t-test). (**d**) Immunofluorescence staining of γH2AX in COV434 and pT&S upon si-FOXP1-mediated gene knockdown. The relative intensity is listed on the right. Data are presented as the mean ± SEM. n = 5 for each group (unpaired two-tailed t-test). (**e**) PCA analysis of three si-NC samples and three si-FOXP1 samples. (**f**) Volcano plot of DEGs. Blue: downregulated DEGs; Red: upregulated DEGs. (**g**) KEGG terms associated with cellular senescence in si-NC group and si-FOXP1 group. P values were calculated by Fisher’s exact test. (**h**) The predicted binding sites for FOXP1 in the promoter of CDKN1A using the JASPAR website.

Cellular senescence is a potential contributor to ovarian aging, particularly in GCs and T&S cells. We employed single-cell regulatory network inference and clustering analysis to identify core transcription factors (TFs) in GCs and T&S cells. TF analysis revealed that *FOXP1*, *SOX4* and *FOS*, which exhibited decreased expression in both cell types, may play crucial roles in ovarian aging (Fig. 6a,b). Subsequently, we used small interfering RNAs (siRNAs) to knock down *FOXP1*, *SOX4* and *FOS* in a human GC line (COV434) to investigate their impact on ovarian aging. Transfection with si-FOXP1 in COV434 led to a notable increase in the expression of senescence-associated genes (Extended Data Fig. 8a).

FOXP1 expression in human ovaries showed an age-related decrease in hGCs and pT&S (Fig. 6c). This decline was confirmed in aged ovaries through western blot and IHC (Fig. 6d,e). Using siRNAs, we silenced FOXP1 in COV434 and pT&S cells, leading to increased senescence-associated (SA)-β-gal staining (Fig. 6f). Furthermore, proteins associated with cellular senescence and DNA damage were upregulated after FOXP1 knockdown (Fig. 6g and Extended Data Fig. 8b). FOXP1 depletion also reduced the numbers of 5-ethynyl-2′-deoxyuridine (EdU)-positive and Ki67-positive cells (Fig. 6h and Extended Data Fig. 8c), suggesting impaired cell proliferation. Enhanced γH2AX fluorescence intensity in COV434 and pT&S cells after FOXP1 silencing indicated activation of the DNA damage response (Extended Data Fig. 8d). These findings suggest a potential association between age-related FOXP1 reduction and senescence in both GCs and T&S cells.

Bulk RNA-seq analysis in FOXP1-knockdown COV434 cells revealed 409 upregulated and 254 downregulated genes (Extended Data Fig. 8e,f). GO analysis indicated enrichment of upregulated genes in ‘response to stimuli’ and ‘immune response’, whereas downregulated genes were associated with ‘cellular developmental processes’ and ‘cellular differentiation’ (Fig. 6i). Notably, senescence markers (*CDKN1A*, *CDKN2A* and *CDKN2B*) and SASPs (*IL-6*, *TNF* and *CXCL8*) were elevated in the si-FOXP1 groups, whereas cell cycle-related genes (*CCNA1*, *CCNB1* and *CCND1*) decreased (Fig. 6j). Si-FOXP1 also increased expression in pathways linked to cellular senescence and immune inflammation, suggesting that FOXP1 silencing induces GCs senescence at the transcriptomic level.

To explore the regulatory mechanism, we focused on FOXP1’s impact on the CDKN1A locus. FOXP1-binding sites within the CDKN1A promoter were identified through promoter occupancy analysis (Extended Data Fig. 8h). Chromatin immunoprecipitation (ChIP)–qPCR confirmed FOXP1 binding to the CDKN1A promoter (Fig. 6k), suggesting that FOXP1 inhibits ovarian cellular senescence by repressing CDKN1A transcription. Dual-luciferase reporter assays demonstrated that FOXP1 activation decreased the activity of the wild-type (WT) CDKN1A promoter, and mutating FOXP1 in the CDKN1A promoter 1535–1573 abrogated this effect (Fig. 6l). Lentivirus-mediated FOXP1 upregulation in COV434 reduced SA-β-gal staining and CDKN1A expression (Fig. 6m–p). Together, these results suggest that FOXP1 inhibits CDKN1A transcription by binding to its promoter, providing insight into the mechanism underlying FOXP1’s role in preventing ovarian cellular senescence.

### FOXP1 knockout in GCs accelerates ovarian aging

**Fig. 7:**
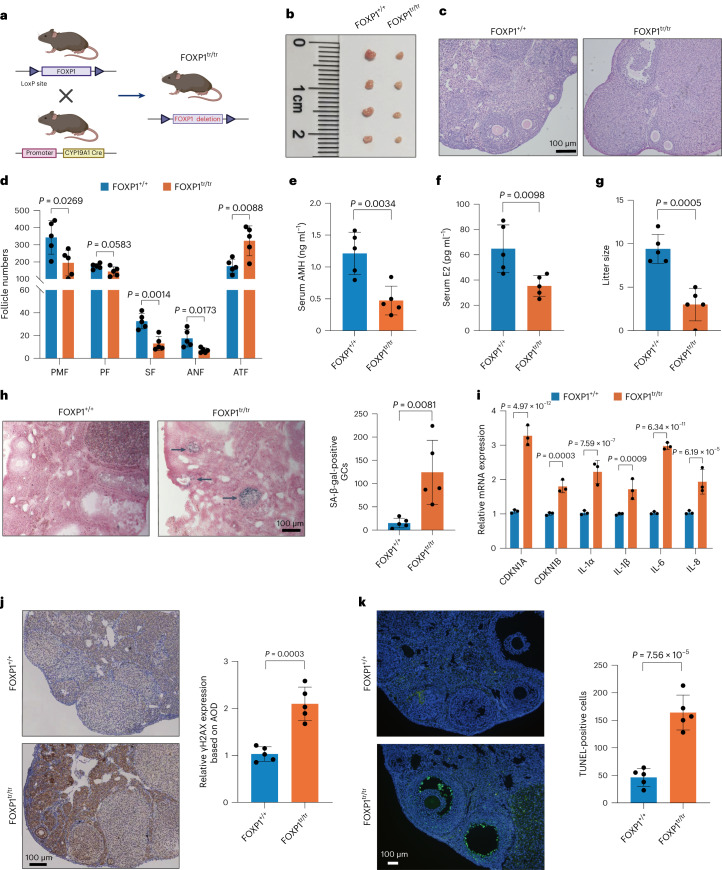
Conditional knockout of FOXP1 in GCs accelerates ovarian aging in mice. **a**, Schematic representation of the deletion of FOXP1 in GCs by using CYP19A1-Cre transgenic mice. **b**, Ovaries of FOXP1^+/+^ and FOXP1^tr/tr^ mice at 12 weeks. **c**, H&E staining of ovaries from FOXP1^+/+^ and FOXP1^tr/tr^ mice. **d**, Comparison of follicle numbers of FOXP1^+/+^ and FOXP1^tr/tr^ mice. Data are presented as the mean ± s.e.m. *n* = 5 for each group (unpaired two-tailed *t*-test). **e**, Serum AMH level of FOXP1^+/+^ and FOXP1^tr/tr^ mice. Data are presented as the mean ± s.e.m. *n* = 5 for each group (unpaired two-tailed *t*-test). **f**, Serum E2 level of FOXP1^+/+^ and FOXP1^tr/tr^ mice. Data are presented as the mean ± s.e.m. *n* = 5 for each group (unpaired two-tailed t-test). **g**, Litter size of mated mice. Data are presented as the mean ± s.e.m. *n* = 5 for each group (unpaired two-tailed *t*-test). **h**, SA-β-gal staining of ovaries from FOXP1^+/+^ and FOXP1^tr/tr^ mice. The number of SA-β-gal-positive cells was shown on the right. Data are presented as the mean ± s.e.m. *n* = 5 for each group (unpaired two-tailed *t*-test). **i**, Relative RNA expression of SASPs in GCs from FOXP1^+/+^ and FOXP1^tr/tr^ mice. Data are presented as the mean ± s.e.m. *n* = 3 for each group (unpaired two-tailed *t*-test). **j**, Representative images of γH2AX staining in ovaries of FOXP1^+/+^ and FOXP1^tr/tr^ mice. Data are presented as the mean ± s.e.m. *n* = 5 for each group (unpaired two-tailed *t*-test). **k**, TUNEL staining of ovaries from FOXP1^+/+^ and FOXP1^tr/tr^ mice. Data are presented as the mean ± s.e.m. *n* = 5 for each group (unpaired two-tailed *t*-test). Source data

To investigate the impact of FOXP1 on ovarian aging, we eliminated the FOXP1 gene from mouse GCs by breeding FOXP1^loxP/loxP^ mice with CYP19A1-Cre transgenic mice (Fig. 7a). At 12 weeks, the ovaries of FOXP1^loxP/loxP^, CYP19A1-Cre (+) (FOXP1^tr/tr^) mice were significantly smaller than those of WT (FOXP1^+/+^) mice (Fig. 7b). Histological and morphometric analyses revealed a reduction in healthy follicle numbers and an increase in atretic follicle numbers in FOXP1^tr/tr^ mice compared to FOXP1^+/+^ mice (Fig. 7c,d). The FOXP1^tr/tr^ females showed reduced serum AMH and estradiol (E2) levels (Fig. 7e,f), indicating a diminished ovarian reserve. Furthermore, litter sizes in FOXP1^tr/tr^ females were smaller to those of littermate controls (Fig. 7g). These findings demonstrate that FOXP1 deletion in GCs expedites ovarian aging, manifested by decreased follicle numbers and reduced offspring sizes.

In the mouse model, the knockout of FOXP1 in GCs led to increased SA-β-gal activity (Fig. 7h). Analysis of cellular senescence-related genes *(CDKN1A*, *CDKN2A*, *IL-1α*, *IL-1β*, *IL-6* and *IL-8*) in WT and FOXP1^tr/tr^ mice ovaries showed a significant increase in mRNA levels in FOXP1^tr/tr^ ovaries, indicating the potential role of FOXP1 in cellular senescence (Fig. 7i). Subsequent examination of γH2AX expression by IHC revealed a significant increase (Fig. 7j). Consistent with this, TUNEL staining demonstrated a higher number of apoptotic GCs in the FOXP1^tr/tr^ group compared to the FOXP1^+/+^ group (Fig. 7k). Overall, these findings suggest that FOXP1 knockout leads to a reduction in ovarian follicular reserve.

### Quercetin protects the ovarian reserve in middle-aged mice

**Fig. 8:**
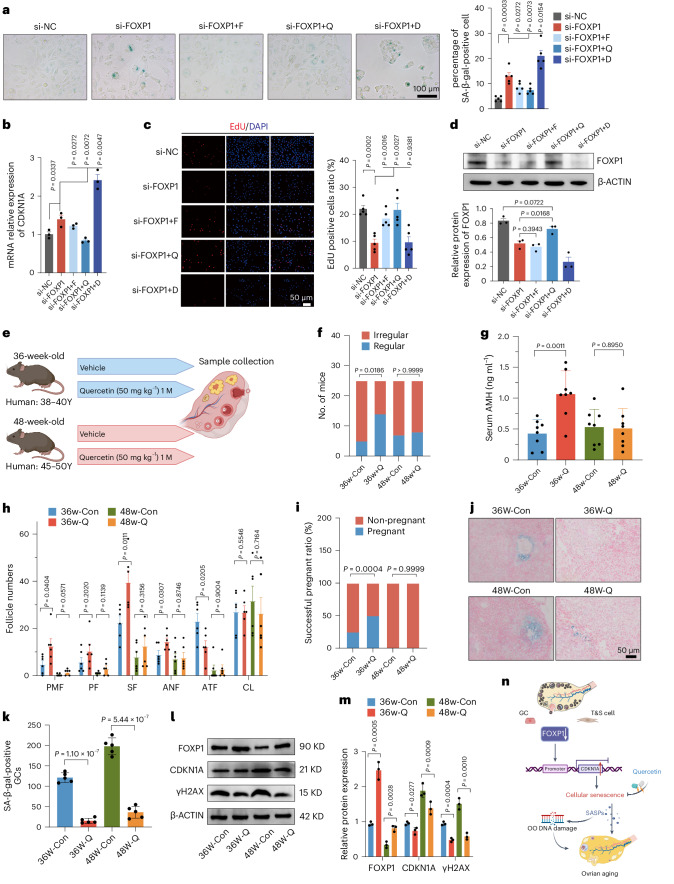
Quercetin treatment protects the ovarian reserve in middle-aged mice. **a**, SA-β-gal staining in COV434 upon administration of fisetin (F), quercetin (Q) and dasatinib (D) in cells with knockdown of FOXP1. Data are presented as the mean ± s.e.m. *n* = 5 for each group (unpaired two-tailed *t*-test). **b**, Relative RNA expression of CDKN1A in si-FOXP1 COV434 treated with F, Q and D. Data are presented as the mean ± s.e.m. *n* = 3 for each group (unpaired two-tailed *t*-test). **c**, EdU incorporation assay of COV434 upon administration of F, Q and D in cells with knockdown of FOXP1. Data are presented as the mean ± s.e.m. *n* = 5 for each group (unpaired two-tailed *t*-test). **d**, FOXP1 protein expression in COV434 treated by F, Q and D. This test was repeated three times. Data are presented as the mean ± s.e.m. (one-way ANOVA). *n* = 3 for each group. **e**, Experimental design to test the effects of quercetin on ovarian aging. **f**, Estrous cycles monitoring of mice. *n* = 25 for each group (Fisher Freeman Halton, two-sided). **g**, Serum AMH levels. Data are presented as the mean ± s.e.m. *n* = 8 for each group (unpaired two-tailed *t*-test). **h**, Follicle counting results according to ovary serial sections. ANF, antral follicle; ATF, atretic follicle; PMF, primordial follicle; PF, primary follicle; SF, secondary follicle; CL, corpus luteum. Data are presented as the mean ± s.d. *n* = 6 for each group (unpaired two-tailed *t*-test). **i**, The proportion of successful pregnant mice. *n* = 8 for each group (chi-squared test, two-sided). **j**, SA-β-gal staining of mice ovaries. **k**, Statistical analysis of SA-β-gal-positive GCs in mice ovaries. Data are presented as the mean ± s.e.m. (one-way ANOVA). *n* = 5 for each group. **l**, Western blot analysis of FOXP1, CDKN1A and γH2AX levels from the ovaries of treated mice. **m**, Densitometry quantified data of western blot analysis. Data are presented as the mean ± s.e.m. (unpaired two-tailed *t*-test). This test was repeated three times. **n**, Schematic illustration showing that downregulation of FOXP1 in GCs and T&S cells contributes to ovarian senescence. Source data

**Extended Data Fig. 9:**
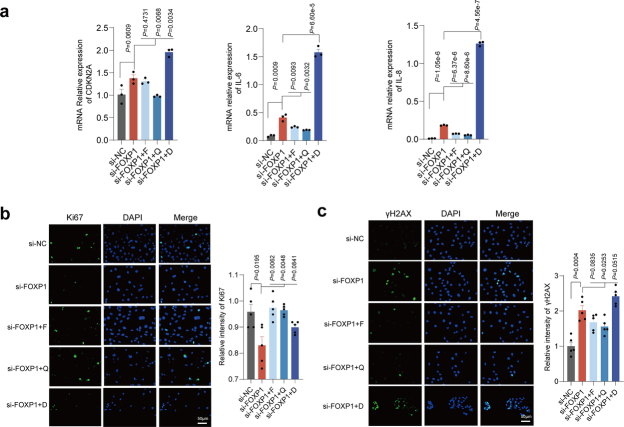
The effect of quercetin in COV434 with knockdown of FOXP1. (**a**) mRNA relative expression of CDKN2A, IL-6 and IL-8 in si-FOXP1 COV434 treated with fisetin (F), quercetin (Q) and dasatinib (D). Data are presented as the mean ± SEM. n = 3 for each group (unpaired two-tailed t-test). (**b**) Immunofluorescence staining of Ki67 upon administration of fisetin (F), quercetin (Q) and dasatinib (D) in COV434 with knockdown of FOXP1. The relative intensity is listed on the right. Data are presented as the mean ± SEM. n = 5 for each group (unpaired two-tailed t-test). (**c**) Immunofluorescence staining of γH2AX upon administration of fisetin (F), quercetin (Q) and dasatinib (D) in COV434 with knockdown of FOXP1. The relative intensity is listed on the right. Data are presented as the mean ± SEM. n = 5 for each group (unpaired two-tailed t-test).

In the pursuit of drugs against ovarian aging, three well-known anti-aging agents (fisetin, quercetin and dasatinib) were investigated^24^. Notably, quercetin and fisetin delayed FOXP1 gene silencing-induced cellular senescence in COV434 cells (Fig. 8a). Quercetin significantly reduced *CDKN1A*, *CDKN2A*, *IL-6* and *IL-8* mRNA levels (Fig. 8b and Extended Data Fig. 9a). EdU staining indicated improved COV434 cell proliferation rates with fisetin and quercetin (Fig. 8c and Extended Data Fig. 9b). Immunofluorescence staining showed decreased γH2AX levels after fisetin or quercetin treatment (Extended Data Fig. 9c). Western blot experiments confirmed that quercetin activated FOXP1 expression (Fig. 8d). These findings suggest that quercetin inhibits GC senescence.

**Extended Data Fig. 10:**
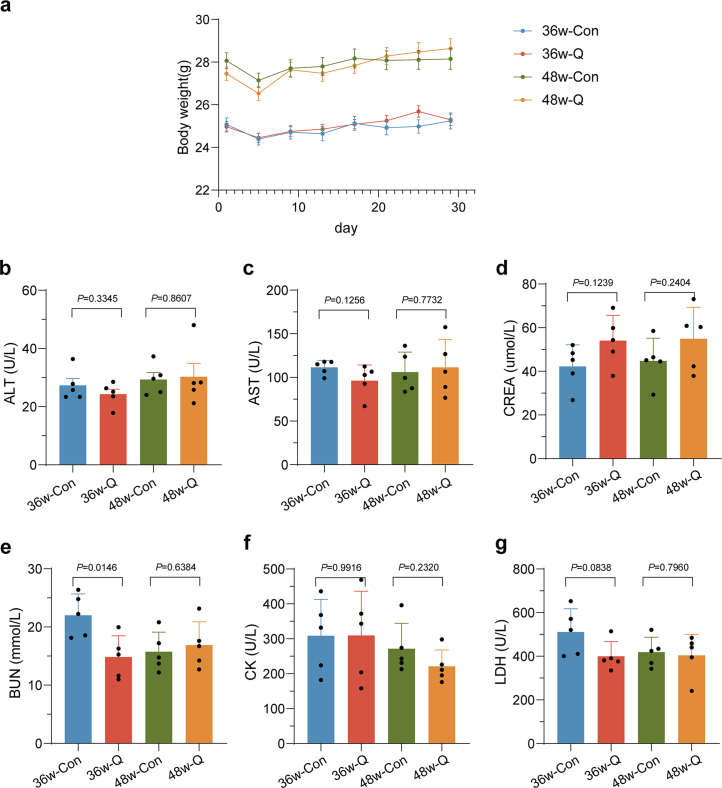
Biological safety evaluation of quercetin treatment in mice. (**a**) The body weight of mice in four groups. Data are presented as the mean ± SEM. n = 25 for each group (unpaired two-tailed t-test). (**b**) and (**c**) The serum level of alanine aminotransferase (ALT) and aspartate transaminase (AST) for liver function. Data are presented as the mean ± SEM. n = 5 for each group (unpaired two-tailed t-test). (**d**) and (**e**) The serum level creatinine (CREA) and blood urea nitrogen (BUN) for renal function. Data are presented as the mean ± SEM. n = 5 for each group (unpaired two-tailed t-test). (**f**) and (**g**) The serum level of creatine kinase (CK) and lactic dehydrogenase (LDH) for heart function. Data are presented as the mean ± SEM. n = 5 for each group (unpaired two-tailed t-test).

To investigate the potential delay of ovarian aging, reproductively aged mice were treated with quercetin. Specifically, 36-week-old and 48-week-old female mice, equivalent to 38–40 and 48–50 years of age in humans, received oral quercetin for 1 month (Fig. 8e). The quercetin-treated group showed regular weight (Extended Data Fig. 10a) and organ function (Extended Data Fig. 10b–g), indicating no side effects. After 1 month, the estrous cycle and serum AMH levels confirmed a protective effect of quercetin on the ovarian reserve in 36-week-old mice, whereas no effect was observed in 48-week-old mice (Fig. 8f,g). As compared to the control group, the number of primordial follicles and antral follicles were significantly increased after quercetin treatment, whereas the follicle proportion of atretic follicles was significantly decreased in 36-week-old mice (Fig. 8h); however, there were no statistical differences of follicles between the control and treatment group in 48-week-old mice (Fig. 8h). Reproductive capability analysis revealed a higher proportion of successful pregnancies in 36-week-old quercetin-treated mice than in the control group (Fig. 8i). These results suggest that quercetin treatment preserves the ovarian reserve and improves ovarian aging in middle-aged life; however, quercetin showed no ovarian protective effects in 48-week-old mice, possibly due to the limited number of follicles, similar to perimenopausal women.

The intrinsic mechanism of quercetin’s protective effect on the ovarian reserve in females was investigated. Quercetin treatment significantly reduced SA-β-gal activity in the ovary (Fig. 8j,k). Accordingly, reduced levels of the senescence markers CDKN1A and γH2AX proteins were observed in quercetin-treated ovaries (Fig. 8l,m). The expression of FOXP1 in the ovaries also significantly increased after treatment with quercetin (Fig. 8l,m). In summary, these findings suggest that quercetin improves ovarian function in middle-aged mice.

## Discussion

In this study, we conducted a comprehensive analysis of single-cell and spatial transcriptomic maps of human ovaries across different age groups, shedding light on spatial and temporal variations in gene expression during ovarian aging. Based on this dataset, our results provided five noteworthy contributions. First, we delineated gene expression signatures and spatial locations for eight types of human ovarian cells, pinpointing cell type-specific DEGs during ovarian aging. Second, an exploration of age-associated changes in gene expression highlighted the DNA damage response as a biological pathway involved in oocyte aging. Third, integrating scRNA-seq data with ST enabled the identification of three GC subtypes and five T&S cell subtypes in the human ovary, along with changes in transcriptomic features during aging. Fourth, we identified FOXP1 as a potential key transcriptional regulator for ovarian cellular senescence, repressing the transcription of the senescence marker CDKN1A. Fifth, our study revealed that quercetin treatment improved for ovarian reserve in reproductively aged mice. These findings provide new insights into human ovarian aging and present potential targets for the treatment of ovarian aging.

Obtaining critical human tissues for studying ovarian aging poses challenges, resulting in limited research on cellular compositions and gene expression changes in this context. While Wang et al. recently utilized scRNA-seq to create a nonhuman primate cell atlas for ovarian aging, differences between nonhuman primates and humans still exist^8^. Although nonhuman primates are recognized as the best animal model for human studies, many evolutionary differences exist between nonhuman primates and humans^25,26^. Llonch et al. compared the transcriptomes of germinal vesicle and in vitro-matured-stage oocytes but focused only on post-ovulation oocytes, excluding developing oocytes and ovarian somatic cells^27^. Our study addresses the gap by presenting the first comparative atlas of temporal and spatial variability in the human ovary during aging, revealing detailed age-related gene expression alterations in oocytes and ovarian somatic cells at the single-cell level; however, the ovarian samples were collected from patients with cervical or endometrium neoplasms before cancer treatment. Despite confirming healthy ovarian tissue through staining, further data analysis is necessary to eliminate potential tumor-related impacts on ovarian function and biases arising from cancer types.

In characterizing the cellular composition of the human ovary, we identified three GC subtypes and four T&S cell subtypes based on distinctive spatiotemporal transcriptomic molecular signatures. These subtypes exhibit specific spatial locations and fulfill distinct functions within the human ovary. Notably, our analysis delved into the intricate components of ovarian stromal cells, previously undescribed. Two subtypes, T&S cell subtype 1 and 4, were uniquely localized in the ovary medulla and cortex bone, respectively. T&S cell subtypes 2 and 5, involved in follicular development and ovulation, exhibited decreased abundance in the old age group. Additionally, we demonstrated that one myofibroblast-like cell type (subtype 3) plays a role in maintaining vascular function in the human ovary.

In aged ovaries, a prominent observation is the manifestation of features associated with cellular senescence across most cell types. These changes encompass heightened cellular senescence score, SASPs, SA-β-gal activity, lipofuscin accumulation, oxidative protein damage and alterations in molecules linked to cellular senescence pathways. Previous studies indicated that two pathways can initiate and maintain cellular senescence: the p53-p21-pRB and the p16-pRB pathways^28^. Notably, a significant increase in CDKN1A/p21 levels, indicative of a p21 pathway-induced senescence-like phenotype, was observed in eight ovarian cell types, suggesting its presence in aged human ovaries. Histological assessments and transcriptional analyses of mouse ovaries revealed significantly increased CDKN1A expression with advancing age^29^. In line with this finding, endometriosis-associated ovarian aging led to increased CDKN1A levels in GCs^30^. The prolonged presence of senescent cells in the tissue is problematic because they secrete SASPs that trigger local inflammation and spread the senescence phenotype^31^. Numerous studies have associated ovarian aging with a chronic low-grade inflammatory environment^12,32,33^, supported by our findings demonstrating the activation of inflammation-associated signal pathways (NF-κB, NOD-like receptor, IL-17 and FoxO) and increased expression of pro-inflammatory factors (such as IL-1α, IL-1β, IL-6, IL-8, TNF and IFNγ) in aged ovaries. This inflammatory microenvironment in the ovary may induce oocyte damage, as indicated by the activation of the DNA damage system in aged oocytes, suggesting chronic inflammation as a potential confounding factor in ovarian aging.

The molecular network governing the senescence of ovarian cells is not well understood. In this study, our analyses identified FOXP1 as a crucial TF regulating cellular senescence in the ovary. During aging, we noted a decline in FOXP1 expression in ovarian GCs and T&S cells, and its knockdown resulted in induced SA-β-gal activity in these cells. Depletion of FOXP1 in GCs activated cellular senescence-associated gene expression, accelerating ovarian aging in mice. FOXP1, known for controlling cell differentiation, proliferation and development^34–36^, has been implicated in attenuating senescence in mesenchymal stem cells and preventing hypertrophic and senescent phenotypes in cardiomyocytes^37,38^. Collectively, our data propose FOXP1 as a potential target for delaying ovarian aging; however, the study has limitations, such as the inability of FOXP1 knockout mice to fully replicate the aging process of human ovaries due to physiological differences. Further investigation is needed to understand how FOXP1 is downregulated in aged human ovaries, particularly exploring its regulation at the epigenetic level.

Quercetin, a dietary flavonoid found in various fruits and vegetables, is recognized for its anti-inflammatory and antioxidant properties, known to prevent age-associated diseases. Our previous findings have established quercetin as a geroprotective drug in cisplatin-exposed mice, enhancing reproductive span, oocyte quality, fertility, ovarian reserve and hormone secretion^39^. In this study, we discovered its efficacy in improving ovarian function in middle-aged mice. Nevertheless, quercetin failed to demonstrate protective effects in older mice, possibly owing to the restricted number of follicles in 48-week-old mice, mirroring the gradual decline seen in humans leading to menopause^3^. This led to the hypothesis that preventing ovarian cellular senescence from middle age might be a promising strategy to delay ovarian aging. Future research is needed to delve into how quercetin precisely regulates FOXP1 expression in the ovary.

In conclusion, we have mapped the spatiotemporal single-cell transcriptomic landscape of human ovarian aging and identified FOXP1 as deregulated with aging and modulating ovarian reserve. The pharmacological activation of FOXP1, as induced by quercetin, emerges as a promising therapeutic strategy for delaying ovarian aging (Fig. 8n). Our study deepens the understanding of human ovarian aging, providing a valuable resource for investigating potential therapeutic interventions. Moving forward, we aim to explore FOXP1 as a potential target for both the diagnosis and treatment of human ovarian aging.

## Methods

### Human samples and ethical statement

Human ovaries were obtained from participants who underwent oophorectomy due to cervical cancer or carcinoma of the endometrium (Supplementary Table 1). All participants were in the proliferative phase during the operation. They did not use any hormone medications and had not undergone radiotherapy or chemotherapy. Pathologists evaluated the ovarian tissues to exclude tumor metastasis. The sample collection was approved by the local ethics committee (TJ-IRB20210319) and all participants provided informed consent.

### Single cell isolation

Human ovarian tissues were preserved in Miltenyi tissue storage solution at an ice bath and transferred to the laboratory within 2 h. Approximately, a 1-cm³ piece was isolated. The tissue was washed with PBS (0.04% BSA) and cut into 0.1-cm³ sections in 2 mg ml^−1^ IV collagenase. The tube containing IV collagenase and tissues was then oscillated in a water bath at 37 °C for 20 min. After centrifugation at 1,200 rpm for 5 min, the supernatant was discarded and the tissues were resuspended in 0.5% trypsin. The tissues in 0.5% trypsin were further oscillated in a water bath at 37 °C for 10 min. Digestion was stopped with DMEM containing 10% BSA and filtered through a 40-μm cell strainer (Millipore). Subsequently, cells were incubated in red blood cell lysis buffer for 10 min, then centrifuged and resuspended in 100–200 μl PBS containing 0.04% BSA. Overall cell viability, confirmed by trypan blue exclusion (above 85%), resulted in single-cell suspensions, counted using a hemocytometer, and the concentration was adjusted to 700–1,200 cells per μl.

### Single-cell RNA sequencing and analysis

A single-cell suspension for each ovary sample was loaded onto a separate channel of a Chromium 10x Genomics single cell 3′ v3 library chip as per the manufacturer’s protocol. Complementary DNA sequencing libraries were prepared according to the manufacturer’s protocol and sequenced on an Illumina NovaSeq 6000 (2 × 150-bp paired-end reads).

Raw sequence reads in FASTQ format from ovary samples were processed and aligned to the GRCh38 human reference transcriptome (https://www.10xgenomics.com/) using the CellRanger v.4.0.0 pipeline (https://www.10xgenomics.com/) with default parameters. The resulting gene expression matrices were merged using the Seurat package v.3 (ref. ^40^). The preprocessing followed the guidelines provided by the Seurat v.3 tutorial. To account for differences in sequencing depth across samples, we normalized expression values for total UMIs per cell and log transformed the counts using the Seurat Normalize Data function.

### Clustering and identification of cell types

All clustering analyses were conducted following the Seurat v.3 integrated tutorial. Variable genes were identified with the FindVariableFeatures function and 2,000 variable genes were selected for subsequent analysis. The first 20 principal components were used for principal-component analysis (PCA). Use the harmony function to remove batch effects on the data. Clustering was performed using the FindClusters function, which works on a *k*-nearest neighbor graph model with a resolution of 0.5, and displayed in UMAP/*t*-distributed stochastic neighbor embedding plots.

To identify DEGs, we used the Seurat FindMarkers function based on a Wilcox likelihood-ratio test with default parameters, and selected the genes expressed in more than 25% of the cells in a cluster and with an average log(fold change) value greater than 0.25 as DEGs. For the cell type annotation of each cluster, we combined the expression of canonical markers found in the DEGs with knowledge from the literature and displayed the expression of markers of each cell type with Featureplot and violin plots that were generated with the Seurat FeaturePlot/Vlnplot function. The cell type of each cluster was identified by known marker genes. For each cell type, we re-ran the Seurat cluster workflow to identify cell subtypes.

### Gene set score analysis

The ‘AddModuleScore’ function in Seurat was used to calculate module scores for gene expression programs in single cells. First, all the analyzed genes were binned based on the average expression and the control genes were randomly selected from each bin. Then, the average expression value of the gene set was calculated at the single-cell level minus the aggregated expression of the control gene set. Gene sets were obtained from the MSigDB database (https://www.gsea-msigdb.org/gsea/msigdb/) and are listed in Supplementary Table 4.

### Pseudotime analysis

To map the differentiation/conversion of particular cell types, pseudotime trajectory analysis was performed with Monocle2 (v.2.99.3)^41^. To construct the trajectory, the Monocle dispersionTable function was used to select highly variable genes from epidermal cells and to perform dimension-reduction with DDRTree. Finally, the trajectory was visualized by plot_cell_trajectory.

### Transcriptional regulatory network analysis

The transcriptional regulatory network was analyzed by the PYSCENIC workflow using default parameters. TFs of hg38 were used as reference TFs and downloaded from RcisTarget (https://resources.aertslab.org/cistarget/). The gene expression matrix of all cell types was normalized from Seurat as input. First, coexpression modules were identified between TFs and the potential target genes based on the gene expression matrix through grnboost2 module. Second, for each coexpression module, *cis*-regulatory motif enrichment analysis was performed among all potential target genes by ctx module, and only the target genes enriched with the motifs of the corresponding TFs were selected as direct target genes. Each TF and its direct target genes were defined as a regulon. Finally, Regulon specificity scores were calculated for each cell. Networks of the TF modules were visualized by Cytoscape (v.3.8.2).

### Cell–cell communication analysis

The cell–cell interactions between different cell types were evaluated using CellChat (v.1.4.0, R package). CellChat takes gene expression data as user input to model the probability of cell–cell communication by integrating gene expression with the existing database consisting of known interactions between signaling ligands, receptors and their cofactors. In this paper, cell–cell interactions were analyzed individually for different conditions following the default pipeline. Normalized count data from each condition were used to create a CellChat object and the recommended preprocessing functions were applied for the analysis of individual datasets with default parameters. CellChatDB.human was used as the database for inferring cell–cell communication. All categories of ligand–receptor interactions in the database were used in the analysis. Communications involving fewer than 10 cells were excluded.

### Spatial transcriptomic experiment

The ovary was embedded with OPTI-MUM in dry ice and saved in −80 °C. The freezing tissue was tested for RNA quality with RIN > 7.0 (RNA pico, Agilent). The tissue optimization experiment (10x Genomics, Visium Spatial Tissue Optimization, Rev A) was performed with imaging of fluorescence footprint and 8 min was identified as optimum permeabilization time. Human ovarian samples were then processed for full ST experiment as the manufacturer’s instructions (10x Genomics, Visium Spatial, Rev B).

### Spatial transcriptomic data processing

We used Space Ranger v.1.2.1 to process raw fastq files. The Space Ranger output files were then imported into the R environment (v.4.0.5) and analyzed using the R package Seurat v.4.0.1 (https://github.com/satijalab/seurat/). We obtained information about the number of spots, UMIs under each tissue and median/mean genes and reads per spot. SCTransform was used to normalize data followed by function RunPCA and FindNeighbors; the PCA dims were determined by the function ElbowPlot (please see Code Availability for details). The spatial expression of selected genes was visualized using the normalized data. The number of expressed UMIs (nUMIs) and genes (nGene) were visualized with the function SpatialPlot. The spatial expression of selected gene sets from the MSigDB database (https://www.gsea-msigdb.org/gsea/msigdb/) was visualized by R package SPATA2 v.0.1.0 (https://themilolab.github.io/SPATA2/) with function plotSurface2 setting ‘parameters smooth = TRUE’ and ‘smooth_span = 0.2’.

### Cell-type annotation of ST data

To spatially map the human ovary in situ, the combined scRNA-seq datasets were integrated with 10x Visium ST data using the anchor-based integration pipeline in Seurat (v.4.0.1), which allowed the transfer of cell-type annotations from scRNA-seq to ST. The annotated scRNA-seq dataset for the same tissue from the identical person was used as reference dataset. The scRNA-seq dataset was also normalized by SCTransform followed by PCA. Cell-type annotation was performed using the Seurat cell-type annotation pipeline with the function FindTransferAnchors setting parameter ‘normalization.method = SCT’ and function TransferData setting parameters ‘prediction.assay = TRUE’ and ‘dims = 1:30’.

To further split GCs (or T&S cells) into subclusters, spots containing these cells were clustered into several groups according to their cell-type annotation. For the echo group, we filtered the original single-cell reference dataset by cell-type annotation to get a new dataset with the same cell type using the Seurat function subset. The Seurat cell-type annotation pipeline was performed for echo spot groups, respectively with the cells of GCs (or T&S cells) relabeled with the subtype names.

### Spatial cell–cell interaction analysis

To study cell–cell interactions in the slides, we used stlearn v.0.4.12 (https://github.com/BiomedicalMachineLearning/stLearn) to measure ligand–receptor (L–R) coexpression. Selected L–R pairs were applied to the ST results, where the cell types in every spatial spot were annotated. The L–R coexpression data and the spatial adjacency information were then import into the Seurat object for visualization of L–R coexpression score in specific anatomical regions.

### Lipofuscin staining

Sudan black B (Solarbio), 0.15 g, was dissolved in 100 ml 70% ethyl alcohol and stored sealed away from light. Frozen sections of human ovaries were incubated in 1% methanol for 5 min and incubated in PBS twice for 5 min, then 50% and 70% ethyl alcohol for 5 min. Afterwards, filtered Sudan black B dye liquor was dropped into the sections for 5–10 min and washed in 50% ethyl alcohol and then ultrapure water. Finally, sections were incubated with nuclear solid red for 5 min and examined by microscopy.

### Immunofluorescence and IHC

After de-paraffinization and rehydration, antigen retrieval of sections was conducted using EDTA Antigen Repair Buffer (pH 9.0) at 100 °C for 23 min. Then sections were washed in PBS three times for 5 min each. For IHC the sections were incubated in 3% H_2_O_2_ for 30 min at room temperature. After blocking with 5% BSA for 30 min at 37 °C, sections were incubated in primary antibodies at 4 °C overnight. The next day, sections were washed in PBS three times and then incubated in donkey anti-rabbit IgG (H + L) (1:200 dilution, AntGene) for 1 h at 37 °C away from light. Finally, sections were stained with 4,6-diamidino-2-phenylindole (DAPI) for 5 min and examined under the microscope. Antibodies used for immunofluorescence and IHC staining are listed in Supplementary Table 5.

### Multiplex IHC

An Opal 4-Color Manual IHC kit was purchased from Akoya Biosciences. In brief, after de-paraffinization and rehydration, antigen retrieval on sections was conducted using AR6 buffer working solution. After blocking for 10 min, the tissues were covered in primary antibody for 1 h at 37 °C. Then, sections were washed with TBST and incubated in secondary antibody working solution for 10 min. Subsequently, the opal fluorophore working solution was applied to tissues for 10 min. To label other primary antibodies, antigen retrieval on sections was conducted using AR6 buffer working solution again and the following steps were performed in sequence as mentioned above. Finally, sections were incubated in DAPI working solution for 5 min and then examined under the microscope. Antibodies used for multiplex IHC staining are listed in Supplementary Table 5.

### RNA isolation and RT–qPCR

Total RNA was exacted with TRIzol Reagent (Invitrogen). After assessing the concentration of RNA with a Nanodrop 2000 ultra-microspectrophotometer (Thermo), 2 μg total RNA was reversed as cDNA using a HiScript II Q RT SuperMix for qPCR (+gDNA wiper) kit. Quantitative PCR with reverse transcription (RT–qPCR) was conducted with ChamQ Universal SYBR qPCR master mix (Vazyme, R223-01, Q711-02) on the CFX96 real-time PCR system (Bio-Rad). All data were calculated by the 2^ΔΔCt^ method. Primers are listed in Supplementary Table 5.

### Western blot analysis

Proteins were extracted from cells and human ovarian tissues. After quantification using a BCA kit, 25 μg protein per sample was used for western blot analysis. In brief, proteins were separated by 10% SDS–PAGE gel and then transferred to polyvinylidene fluoride (PVDF) membranes. After blocking with 5% BSA for 1 h at room temperature, blots were incubated with specific primary antibodies overnight at 4 °C. The primary antibodies are listed in Supplementary Table 5. The next day, blots were rewarmed for 1 h and incubated with secondary antibodies for 1 h. Finally, a ChemiDoc TMXRS+ system was used for image acquisition.

### Isolation of human GCs

After oocyte retrieval, follicular fluid (FF) was collected from women of different ages who received in vitro fertilization because of male infertility. The GCs were isolated from the FF. The FF was centrifuged at 2,000 rpm for 10 min and pellets were resuspended in 1 ml PBS. Then the above cell suspension was added to 4 ml 50% Percoll solution softly without disturbing the liquid level to purify the hGCs. After centrifugation at 400*g* for 30 min, with acceleration at zero, pellets were collected from the middle liquid level. After incubation in red blood cell lysis buffer, hGCs were stored at −80 °C until RNA extraction.

### Isolation and culture of pT&S cells

pT&S cells were isolated from women of different ages as previously mentioned^42^. Then the pT&S cells were seeded into six-well plates and cultured through 2–3 population doublings in DMEM containing 5% fetal bovine serum (FBS), 5% horse serum, 20 nM insulin, 20 nM selenium, 1.0 μM vitamin E, penicillin (100 IU ml^−1^) and streptomycin (0.1 mg ml^−1^). Cells were maintained in an environment of 5% CO_2_ at 37 °C.

### Cell culture and treatment

COV434 cells and primary pT&S cells were transfected with siRNA (RIBOBIO) targeting FOXP1, SOX4 and FOS using Lipofectamine 3000 (Thermo, L3000015). The sequences of the siRNAs are listed in Supplementary Table 5. In brief, cells were incubated in 50 nM siRNAs for 6 h and cultured in DMEM containing 10% FBS for another 48 h. For exploring senolytics fighting ovarian cell senescence, cells were treated with quercetin (10 μM, MedChemExpress), dasatinib (100 nM, MedChemExpress) or fisetin (10 μM, MedChemExpress) for 24 h. After that, cells were used for RT–qPCR, western blot, SA-β-gal staining and immunofluorescence.

### SA-β-gal staining

For SA-β-gal staining, we used a Senescence β-Galactosidase Staining kit (Beyotime Biotechnology) or Senescence Green Detection kit (Invitrogen, C10850). In brief, cells or ovarian tissue were washed with PBS and incubated in stationary liquid for 15 min at room temperature. After washing in PBS three times for 3 min each, cells were incubated in dyeing working fluid for 14 h (Senescence β-Galactosidase Staining kit) or 2 h (Senescence Green Detection kit) in 37 °C without CO_2_. The next day, dyeing working fluid was discarded and washed with PBS three times. Finally, the sections were examined under the microscope.

### ChIP

ChIP was conducted with COV434 cells using the SimpleChIP Enzymatic Chromatin IP kit (Magnetic Beads) (Cell Signaling Technology). In brief, chromatin was crosslinked by 1% formaldehyde and digested with micrococcal nuclease. FOXP1 antibody, normal rabbit IgG and Protein G Magnetic Beads were used to precipitate specific chromatin fragments. DNA fragments were purified using DNA purification spin columns after reverse crosslinking of the DNA–protein complex. Enrichment of DNA sequences was determined by RT–qPCR. The primers used for ChIP–qPCR are listed in Supplementary Table 5.

### Generation of GC-conditional FOXP1 knockout mice

Mice with the targeted FOXP1 mutation and CYP19A1-Cre knock-in mice on a C57BL/6J background were generated by Shanghai Model Organisms. Initially, CYP19A1-Cre heterozygotes were bred with FOXP1^flox/flox^ mice. The resulting male FOXP1^flox/+^, CYP19A1-Cre mice were bred to female Cre (−), FOXP1^flox/flox^ mice to generate FOXP1^flox/flox^, CYP19A1-Cre males and females. For the final cross, FOXP1^flox/flox^, CYP19A1-Cre males were bred with FOXP1^flox/flox^, Cre (−) females to generate FOXP1^flox/flox^, CYP19A1-Cre females. FOXP1^flox/flox^, CYP19A-Cre (−) female littermates served as controls.

### Quercetin administration in mice

All of the animal protocols and experiments procedures used in this study were approved by the Experimental Animal Committee of Tongji Hospital (TJH202304003). The 36-week-old and 48-week-old C57BL/6 mice were purchased from Beijing Huafukang. The mice were raised in the Tongji Hospital under specific-pathogen-free conditions with a 12-h light–dark cycle and free access to food and water at 25 °C. The mice were randomly divided into the control group and the treatment group. The mice in the treatment group were given 50 mg kg^−1^ quercetin (MedChemExpress, HY-18085, diluted with 0.5% CMC-Na) or the equivalent volume of solvent by oral gavage every 3 days for 1 month.

### Follicle counts

The ovaries were serially sectioned at 5-μm thickness and stained with hematoxylin and eosin (H&E). The primordial, primary, secondary and antral follicles were identified as previously reported^43^. Follicles were counted in sections at least 25 µm apart (each fifth section) spanning the entire ovary.

### Serum hormone testing

Human or mice whole-blood samples were centrifuged to collect the serum. Assays for AMH and E2 levels were performed following the manual instructions of the ELISA kits (CUSABIO).

### Estrous cycle examination

The pipette with 20 µl saline was gently inserted into the vaginal canal. Then, the vaginal fluid was smeared on slides, stained with H&E and observed under a light microscope. The estrous cycle was classified into four stages, including the proestrus, estrus, metestrus and diestrus phases^44^.

### Statistical analyses

Data in the bar plots are shown as the mean ± s.e.m. All experimental data were analyzed using unpaired *t*-tests or one-way analysis of variance (ANOVA) to compare differences between groups (GraphPad v.9.0 Software). *P* values <0.05 were considered to be statistically significant. Blank indicates not significant. A Spearman’s rank correlation coefficient (*r*) was used to calculate the correlation-associated statistical significance in GraphPad v.9.0.

### Reporting summary

Further information on research design is available in the Nature Portfolio Reporting Summary linked to this article.

### Supplementary information

Reporting Summary

Supplementary Table 1 Information of the human ovarian samples for scRNA-seq and ST-seq.

Supplementary Table 2 Markers of each cell type.

Supplementary Table 3 List of DEGs of scRNA-seq.

Supplementary Table 4 List of genes.

Supplementary Table 5 Antibodies and sequences for RT–PCR, siRNA.

### Source data

Source Data Fig. 6 Unprocessed western blots.

Source Data Fig. 8 Unprocessed western blots.

Source Data Extended Data Fig. 3 Unprocessed western blots.

Source Data Statistical Source Data.

## Data Availability

The raw data of scRNA sequencing and ST presented in this study have been deposited into the Gene Expression Omnibus database under accession code GSE255690. Any other data underlying this study will be provided by the corresponding authors upon reasonable request. Source data are provided with this paper.

## Code availability

The analysis code for scRNA sequencing and ST is available on GitHub at https://zenodo.org/doi/10.5281/zenodo.10867453 (ref. ^45^).

## References

[1] Baerwald AR, Adams GP, Pierson RA (2012). Ovarian antral folliculogenesis during the human menstrual cycle: a review. Hum. Reprod. Update.

[2] Broekmans FJ, Knauff EA, te Velde ER, Macklon NS, Fauser BC (2007). Female reproductive ageing: current knowledge and future trends. Trends Endocrinol. Metab..

[3] Broekmans FJ, Soules MR, Fauser BC (2009). Ovarian aging: mechanisms and clinical consequences. Endocr. Rev..

[4] Hsueh AJ, Kawamura K, Cheng Y, Fauser BC (2015). Intraovarian control of early folliculogenesis. Endocr. Rev.

[5] Tabula Muris Consortium. (2020). A single-cell transcriptomic atlas characterizes ageing tissues in the mouse. Nature.

[6] Fan X (2019). Single-cell reconstruction of follicular remodeling in the human adult ovary. Nat. Commun..

[7] Wagner M (2020). Single-cell analysis of human ovarian cortex identifies distinct cell populations but no oogonial stem cells. Nat. Commun..

[8] Wang S (2020). Single-Cell transcriptomic atlas of primate ovarian aging. Cell.

[9] Russ JE, Haywood ME, Lane SL, Schoolcraft WB, Katz-Jaffe MG (2022). Spatially resolved transcriptomic profiling of ovarian aging in mice. iScience.

[10] Zhang T (2019). Mitochondrial dysfunction and endoplasmic reticulum stress involved in oocyte aging: an analysis using single-cell RNA-sequencing of mouse oocytes. J. Ovarian Res..

[11] Wei Y (2023). Single-cell profiling of mouse and primate ovaries identifies high levels of EGFR for stromal cells in ovarian aging. Mol. Ther. Nucleic Acids.

[12] Ben Yaakov T, Wasserman T, Aknin E, Savir Y (2023). Single-cell analysis of the aged ovarian immune system reveals a shift towards adaptive immunity and attenuated cell function. eLife.

[13] Isola JVV (2024). A single-cell atlas of the aging mouse ovary. Nature Aging.

[14] Wei Y (2023). Single-cell transcriptome analysis of the mouse and primate ovaries reveals oocyte-specific expression patterns of risk genes in ovarian aging. MedComm.

[15] Eng CL (2019). Transcriptome-scale super-resolved imaging in tissues by RNA seqFISH. Nature.

[16] Chen KH, Boettiger AN, Moffitt JR, Wang S, Zhuang X (2015). RNA imaging. Spatially resolved, highly multiplexed RNA profiling in single cells. Science.

[17] Salmen F (2018). Barcoded solid-phase RNA capture for spatial transcriptomics profiling in mammalian tissue sections. Nat. Protoc..

[18] Secomandi L, Borghesan M, Velarde M, Demaria M (2022). The role of cellular senescence in female reproductive aging and the potential for senotherapeutic interventions. Hum. Reprod. Update.

[19] Zhang Y (2018). Transcriptome landscape of human folliculogenesis reveals oocyte and granulosa cell interactions. Mol. Cell.

[20] Kadomatsu K, Kishida S, Tsubota S (2013). The heparin-binding growth factor midkine: the biological activities and candidate receptors. J. Biochem..

[21] Peng JY (2016). Molecular cloning, expression analysis, and function of decorin in goat ovarian granulosa cells. Domest. Anim. Endocrinol..

[22] Kedem A (2020). Elucidating Decorin’s role in the preovulatory follicle. J. Ovarian Res..

[23] Oria RB, de Almeida JZ, Moreira CN, Guerrant RL, Figueiredo JR (2020). Apolipoprotein E Effects on Mammalian Ovarian Steroidogenesis and Human Fertility. Trends Endocrinol. Metab..

[24] Di Micco R, Krizhanovsky V, Baker D, d’Adda di Fagagna F (2021). Cellular senescence in ageing: from mechanisms to therapeutic opportunities. Nat. Rev. Mol. Cell Biol..

[25] Rogers J, Gibbs RA (2014). Comparative primate genomics: emerging patterns of genome content and dynamics. Nat. Rev. Genet..

[26] Zhang ZD, Frankish A, Hunt T, Harrow J, Gerstein M (2010). Identification and analysis of unitary pseudogenes: historic and contemporary gene losses in humans and other primates. Genome Biol..

[27] Llonch S (2021). Single human oocyte transcriptome analysis reveals distinct maturation stage-dependent pathways impacted by age. Aging Cell.

[28] Riessland M (2019). Loss of SATB1 Induces p21-Dependent cellular senescence in post-mitotic dopaminergic neurons. Cell Stem Cell.

[29] Ansere VA (2021). Cellular hallmarks of aging emerge in the ovary prior to primordial follicle depletion. Mech. Ageing Dev..

[30] Lin X (2020). Excessive oxidative stress in cumulus granulosa cells induced cell senescence contributes to endometriosis-associated infertility. Redox Biol..

[31] Munoz-Espin D, Serrano M (2014). Cellular senescence: from physiology to pathology. Nat. Rev. Mol. Cell Biol..

[32] Xiao Y (2022). Macrophage-derived extracellular vesicles regulate follicular activation and improve ovarian function in old mice by modulating local environment. Clin. Translat. Med..

[33] Huang Y (2019). Affecting premature ovarian insufficiency. J. Immunol. Res..

[34] Wang B (2004). Foxp1 regulates cardiac outflow tract, endocardial cushion morphogenesis and myocyte proliferation and maturation. Development.

[35] Shu W (2007). Foxp2 and Foxp1 cooperatively regulate lung and esophagus development. Development.

[36] Feng X (2011). Transcription factor Foxp1 exerts essential cell-intrinsic regulation of the quiescence of naive T cells. Nat. Immunol..

[37] Li H (2017). FOXP1 controls mesenchymal stem cell commitment and senescence during skeletal aging. J. Clin. Invest..

[38] Zhang Y (2023). Single-nucleus transcriptomics reveals a gatekeeper role for FOXP1 in primate cardiac aging. Protein Cell.

[39] Du D (2022). Senotherapy protects against cisplatin-induced ovarian injury by removing senescent cells and alleviating DNA damage. Oxid. Med. Cell. Longev..

[40] Stuart T (2019). Comprehensive integration of single-cell data. Cell.

[41] Qiu X (2017). Reversed graph embedding resolves complex single-cell trajectories. Nat. Methods.

[42] McAllister JM, Byrd W, Simpson ER (1994). The effects of growth factors and phorbol esters on steroid biosynthesis in isolated human theca interna and granulosa-lutein cells in long term culture. J. Clin. Endocrinol. Metab..

[43] Wu M (2023). Microenvironmentally responsive chemotherapeutic prodrugs and CHEK2 inhibitors self-assembled micelles: protecting fertility and enhancing chemotherapy. Adv. Mater..

[44] Wang F (2022). BNC1 deficiency-triggered ferroptosis through the NF2-YAP pathway induces primary ovarian insufficiency. Nat. Commun..

[45] Sxw3078/Human_Ovary_Aging: first release of Human_Ovary_Aging (v1.0.0). *Zenodo*10.5281/zenodo.10867454 (2024).

