# Supplementary material for: Spatiotemporal transcriptomic changes of human ovarian aging and the regulatory role of FOXP1
Source: Nat Aging. 2024 Apr 9;4(4):527–45. doi: 10.1038/s43587-024-00607-1 (PMC11031396; doi:10.1038/s43587-024-00607-1)
Supplement: Supplementary file 1 — Reporting Summary [file 43587_2024_607_MOESM1_ESM.pdf]

nature portfolio

Corresponding author(s): DAPR.NATAGING-A02186

Last updated by author(s): Jan 10, 2024

Reporting Summary

Nature Portfolio wishes to improve the reproducibility of the work that we publish. This form provides structure for consistency and transparency in reporting. For further information on Nature Portfolio policies, see our Editorial Policies and the Editorial Policy Checklist.

Statistics

For all statistical analyses, confirm that the following items are present in the figure legend, table legend, main text, or Methods section.

n/a	Confirmed	
☐	☒	The exact sample size (*n*) for each experimental group/condition, given as a discrete number and unit of measurement
☐	☒	A statement on whether measurements were taken from distinct samples or whether the same sample was measured repeatedly
☐	☒	The statistical test(s) used AND whether they are one- or two-sided<br>*Only common tests should be described solely by name; describe more complex techniques in the Methods section.*
☐	☒	A description of all covariates tested
☐	☒	A description of any assumptions or corrections, such as tests of normality and adjustment for multiple comparisons
☐	☒	A full description of the statistical parameters including central tendency (e.g. means) or other basic estimates (e.g. regression coefficient) AND variation (e.g. standard deviation) or associated estimates of uncertainty (e.g. confidence intervals)
☐	☒	For null hypothesis testing, the test statistic (e.g. *F*, *t*, *r*) with confidence intervals, effect sizes, degrees of freedom and *P* value noted<br>*Give P values as exact values whenever suitable.*
☒	☐	For Bayesian analysis, information on the choice of priors and Markov chain Monte Carlo settings
☒	☐	For hierarchical and complex designs, identification of the appropriate level for tests and full reporting of outcomes
☐	☒	Estimates of effect sizes (e.g. Cohen's *d*, Pearson's *r*), indicating how they were calculated

Our web collection on statistics for biologists contains articles on many of the points above.

Software and code

Policy information about availability of computer code

Data collection

No open source data were used.

Data analysis

Used R version 4.0.3, RStudio version 1.4.1103, Image Lab 6.0, Image J 1.46r, Bio-Rad CFX Manager, GraphPad Prism 8.0.
Packages used in R were as follows:
R package Monocle2 55 (version 2.99.3)
RcisTarget□https://resources.aertslab.org/cistarget/□
Cellranger v4.0.0
Seurat package v3, https://satijalab.org/seurat/vignettes.html
MSigDB database, https://www.gsea-msigdb.org/gsea/msigdb/
R package Monocle2 version 2.99.3, http://www.bioconductor.org/packages/release/bioc/html/monocle.html
RcisTarget, https://resources.aertslab.org/cistarget/
Cytoscape (version 3.8.2), https://cytoscape.org/
Space Ranger V1.2.1, https://support.10xgenomics.com/spatial-gene-expression/software/pipelines/latest/installation
Stlearn v0.3.2, https://github.com/BiomedicalMachineLearning/stLearn
Metascape version 3.5, http://metascape.org/

For manuscripts utilizing custom algorithms or software that are central to the research but not yet described in published literature, software must be made available to editors and reviewers. We strongly encourage code deposition in a community repository (e.g. GitHub). See the Nature Portfolio guidelines for submitting code & software for further information.

Data

Policy information about availability of data

All manuscripts must include a data availability statement. This statement should provide the following information, where applicable:

- Accession codes, unique identifiers, or web links for publicly available datasets
- A description of any restrictions on data availability
- For clinical datasets or third party data, please ensure that the statement adheres to our policy

The analysis code for single cell RNA-sequencing and spatial transcriptomic is available at https://github.com/Sxw3078/Human_Ovary_Aging/tree/main.

Research involving human participants, their data, or biological material

Policy information about studies with human participants or human data. See also policy information about sex, gender (identity/presentation), and sexual orientation and race, ethnicity and racism.

Reporting on sex and gender	There is no male-female distinction involved in the study. We only studied the ovaries of female in biological attribute.
Reporting on race, ethnicity, or other socially relevant groupings	All samples were deidentified and we do not have access to other information including race, ethnicity, or other socially relevant groupings.
Population characteristics	We obtained ovarian samples for single cell RNA-sequencing and spatial transcriptomic from cognitively normal individuals aged 18, 19, 22, 28, 29 years (young group) and aged 36, 37, 38, 39, 39 years (middle group) and aged 47, 48, 49, 49, 49 years (old group). Please see Supplemental Table 1 for population characteristic information.
Recruitment	Women were consented in person. All women signed informed consents. Samples collected in Tongji Hospital, Tongji Medical College, Huazhong University of Science and Technology were also approved by the local ethics committee (TJ-IRB20210319). Our priority in selecting women for this study was their age. No obvious cancer metastasis was found in all ovarian tissues during operation, and postoperative pathology showed no ovarian metastasis. None of them presented with endocrine related diseases and they received no hormonal induction of their ovaries. Patients donated a part of the ovarian tissue for this research by informed consent.
Ethics oversight	The study was approved by the Ethics Committee in Tongji Hospital, Tongji Medical College, Huazhong University of Science and Technology (TJ-IRB20210319). All women have signed informed consents.

Note that full information on the approval of the study protocol must also be provided in the manuscript.

Field-specific reporting

Please select the one below that is the best fit for your research. If you are not sure, read the appropriate sections before making your selection.

☒ Life sciences ☐ Behavioural & social sciences ☐ Ecological, evolutionary & environmental sciences

For a reference copy of the document with all sections, see nature.com/documents/nr-reporting-summary-flat.pdf

Life sciences study design

All studies must disclose on these points even when the disclosure is negative.

Sample size	No statistical methods were used to pre-determine sample size, but our sample size (n = 9) are similar to those reported in previous publications (Wang, S. et al., Cell, 2020; Fan, X.et al., Nature Communications, 2019; Wagner, M.et al., Nature Communications, 2020;)
Data exclusions	Do data were excluded from the analyses.
Replication	Most of the experiments were conducted three times independently with similar results. Please refer to each figure legend for more details.
Randomization	15 human ovaries used for sequencing were divided into three groups according to their ages. Five women aged 18y, 19y, 22y, 28y, 29y were clarified as the young group. Five women aged 36y, 37y, 38y, 39y, 39y were clarified as the middle group. Five women aged 47y, 48y, 49y, 49y, 49y were clarified as the old group.
Blinding	Investigators were not blinded to group allocation due to the fact that we need to carefully documenting the differences during sample collection, so blinding was not always possible. When possible, data analysis and image acquisition was performed in a blind manner.

Reporting for specific materials, systems and methods

We require information from authors about some types of materials, experimental systems and methods used in many studies. Here, indicate whether each material, system or method listed is relevant to your study. If you are not sure if a list item applies to your research, read the appropriate section before selecting a response.

Materials & experimental systems

n/a	Involved in the study
☐	☒ Antibodies
☐	☒ Eukaryotic cell lines
☒	☐ Palaeontology and archaeology
☐	☒ Animals and other organisms
☒	☐ Clinical data
☒	☐ Dual use research of concern
☒	☐ Plants

Methods

n/a	Involved in the study
☒	☐ ChIP-seq
☒	☐ Flow cytometry
☒	☐ MRI-based neuroimaging

Antibodies

Antibodies used

Cell Signaling Technology, FOXP1 Rabbit Antibody, catalog number #2005, lot number 3.
Proteintech, NLRP3 Rabbit pAb, catalog number, catalog number 19771-1-AP, lot number 00102786.
Cell Signaling Technology, NF-κB p65 (D14E12) XP® Rabbit mAb, catalog number #8242, lot number 8.
Cell Signaling Technology, Phospho-NF-κB p65 (Ser536) (93H1) Rabbit mAb, catalog number #3033, lot number 16.
Abclonal, Phospho-Histone H2AX-S139 Rabbit mAb, catalog number AP0687, 4000000110.
Abclonal, β-Actin Rabbit mAb (High Dilution), catalog number AC026.
Proteintech, P21 Polyclonal antibody, catalog number 10355-1-AP, lot number 00085901.
Abcam, Mouse monoclonal [15A3] to DNA/RNA Damage, catalog number ab62623.
Abclonal, Nitro-Tyrosine Rabbit pAb, catalog number A20506, lot number 3561654104.
Proteintech, AMH Polyclonal antibody,catalog number 14461-1-AP.
Abclonal, GSTA1 Rabbit pAb, catalog number A18266.
Proteintech, ZP3 Polyclonal antibody, catalog number 21279-1-AP.
Abclonal, TUBB8 Rabbit pAb, catalog number A12617.
Santa Cruz Biotechnology, Ki67 antibody, catalog number sc-23900.
AntGene, ALexarFlour 488 Donkey anti Rabbit IgG(H L), ANT023.
AntGene, ALexarFlour 594 Donkey anti Rabbit IgG(H L), ANT029.
Supplemental Table 5 for more information for antibodies.

Validation

All of these antibodies were optimized and used previously in the lab and were used in multiple papers published from our lab such as:
Zhou S, Xi Y, Chen Y, Zhang Z, Wu C, Yan W, Luo A, Wu T, Zhang J, Wu M, Dai J, Shen W, Zhang F, Ding W, Wang S. Ovarian Dysfunction Induced by Chronic Whole-Body PM2.5 Exposure. Small. 2020 Aug;16(33):e2000845. doi: 10.1002/smll.202000845. Epub 2020 Jul 19. PMID: 32686359.
Chen Q, Xu Z, Li X, Du D, Wu T, Zhou S, Yan W, Wu M, Jin Y, Zhang J, Wang S. Epigallocatechin gallate and theaflavins independently alleviate cyclophosphamide-induced ovarian damage by inhibiting the overactivation of primordial follicles and follicular atresia. Phytomedicine. 2021 Nov;92:153752. doi: 10.1016/j.phymed.2021.153752. Epub 2021 Sep 14. PMID: 34601223.
Li M, Zhou S, Wu Y, Li Y, Yan W, Guo Q, Xi Y, Chen Y, Li Y, Wu M, Zhang J, Wei J, Wang S. Prenatal exposure to propylparaben at human-relevant doses accelerates ovarian aging in adult mice. Environ Pollut. 2021 Sep 15;285:117254. doi: 10.1016/j.envpol.2021.117254. Epub 2021 Apr 28. PMID: 33957517.
The resource of antibodies were listed as follows:
SOX4 Rabbit pAb, https://abclonal.com.cn/catalog/A10717
FOXP1 Rabbit Antibody,https://www.cellsignal.cn/products/primary-antibodies/foxp1-antibody/2005?site-search-type=Products&N=4294956287&Ntt=foxp1&fromPage=plp
NLRP3 Rabbit pAb, https://www.ptgcn.com/products/NALP3-Antibody-19771-1-AP.htm
NF-κB p65 (D14E12) XP® Rabbit mAb, https://www.cellsignal.cn/products/primary-antibodies/nf-kb-p65-d14e12-xp-rabbit-mab/8242?site-search-type=Products&N=4294956287&Ntt=nf-kb&fromPage=plp
Phospho-NF-κB p65 (Ser536) (93H1) Rabbit mAb, https://www.cellsignal.cn/products/primary-antibodies/phospho-nf-kb-p65-ser536-93h1-rabbit-mab/3033?site-search-type=Products&N=4294956287&Ntt=3033&fromPage=plp&_requestid=1916665
Phospho-Histone H2AX-S139 Rabbit mAb, https://abclonal.com.cn/catalog/AP0687
β-Actin Rabbit mAb (High Dilution), https://abclonal.com.cn/catalog/AC026
P21 Polyclonal antibody, https://www.ptgcn.com/products/P21-Antibody-10355-1-AP.htm
Mouse monoclonal [15A3] to DNA/RNA Damage,https://www.abcam.cn/dnarna-damage-antibody-15a3-ab62623.html
Nitro-Tyrosine Rabbit pAb, https://abclonal.com.cn/catalog/A20506
AntGene, ALexarFlour 488 Donkey anti Rabbit IgG(H L),http://antgene.cn/pd.jsp?id=30923#skeyword=488&_pp=0_35

Eukaryotic cell lines

Policy information about cell lines and Sex and Gender in Research

Cell line source(s)	COV434 cell lines were purchased from Procell.
Authentication	COV434 cell lines were authenticated by China Center for Type Culture Collection

Mycoplasma contamination	All cell lines were teated negative for mycoplasma contamination.
Commonly misidentified lines (See ICLAC register)	There is no misidentified lines in this study.

Animals and other research organisms

Policy information about studies involving animals; ARRIVE guidelines recommended for reporting animal research, and Sex and Gender in Research

Laboratory animals	For quercetin administration, mice used were female C57BL/6 mice from Beijing Huafukang. For granulosa cell-conditional FOXP1 knockout mice, mice with the targeted FOXP1 mutation and CYP19A1-Cre knock-in mice on a C57BL/6 J background were generated by the Shanghai Model Organisms (China).The mice were raised in the Tongji Hospital under specific pathogen-free (SPF) conditions with a 12-h light/dark cycle and free access to food and water at 25°C.
Wild animals	No wild animals were used in the study.
Reporting on sex	Our research on ovarian function necessitated the selection of female mice as the primary subjects. However, for the experiment involving the cohabitation and breeding of mice, male mice were utilized. The rationale behind this choice was twofold: female mice offer a precise representation for ovarian studies, and the introduction of male mice in the breeding experiment was imperative to create a realistic breeding setting. This approach, therefore, ensured that the research findings would be both authentic and relevant.
Field-collected samples	No field-collected samples were used in the study.
Ethics oversight	All of the animal protocols and experiments procedures used in this study were approved by the Experimental Animal Committee of Tongji Hospital (TJH202304003).

Note that full information on the approval of the study protocol must also be provided in the manuscript.

Plants

Seed stocks	*Report on the source of all seed stocks or other plant material used. If applicable, state the seed stock centre and catalogue number. If plant specimens were collected from the field, describe the collection location, date and sampling procedures.*
Novel plant genotypes	*Describe the methods by which all novel plant genotypes were produced. This includes those generated by transgenic approaches, gene editing, chemical/radiation-based mutagenesis and hybridization. For transgenic lines, describe the transformation method, the number of independent lines analyzed and the generation upon which experiments were performed. For gene-edited lines, describe the editor used, the endogenous sequence targeted for editing, the targeting guide RNA sequence (if applicable) and how the editor was applied.*
Authentication	*Describe any authentication procedures for each seed stock used or novel genotype generated. Describe any experiments used to assess the effect of a mutation and, where applicable, how potential secondary effects (e.g. second site T-DNA insertions, mosiacism, off-target gene editing) were examined.*
