# Supplementary figures and images for: Spatiotemporal transcriptomic changes of human ovarian aging and the regulatory role of FOXP1
Source: Nat Aging. 2024 Apr 9;4(4):527–45. doi: 10.1038/s43587-024-00607-1 (PMC11031396; doi:10.1038/s43587-024-00607-1)

Fig. 6e

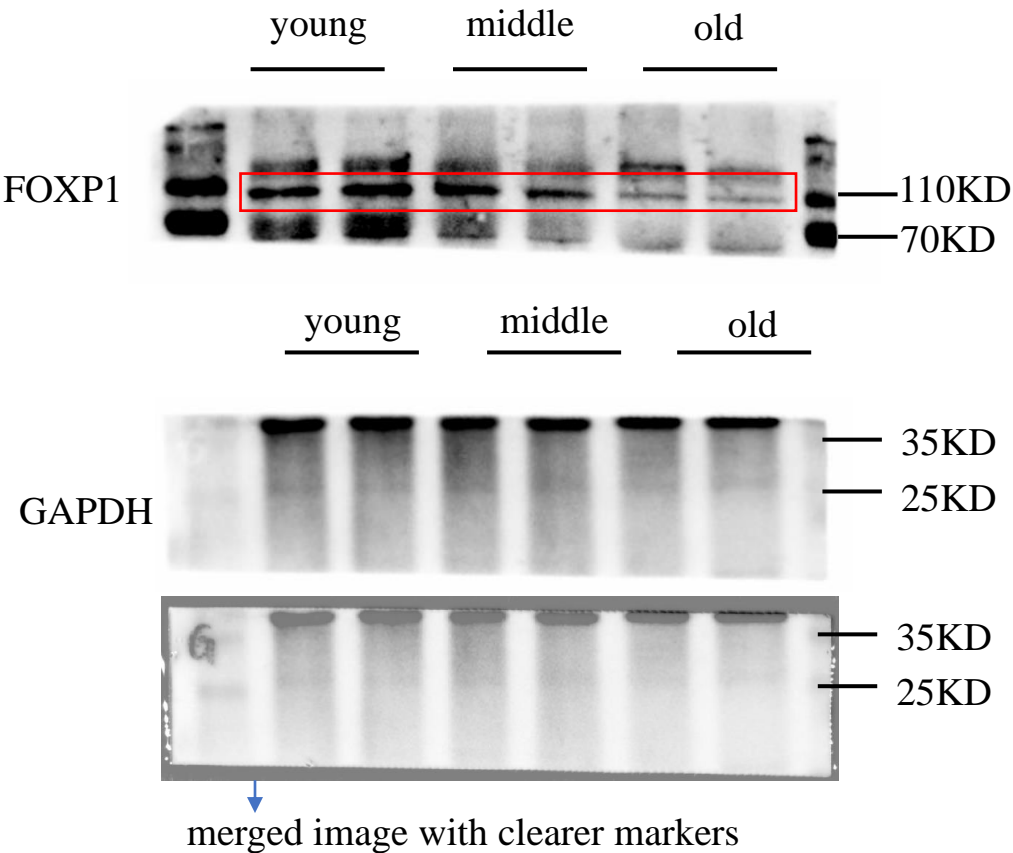

Fig. 6g COV434

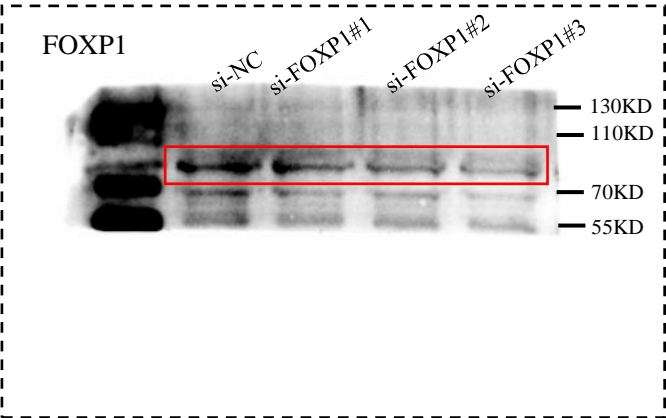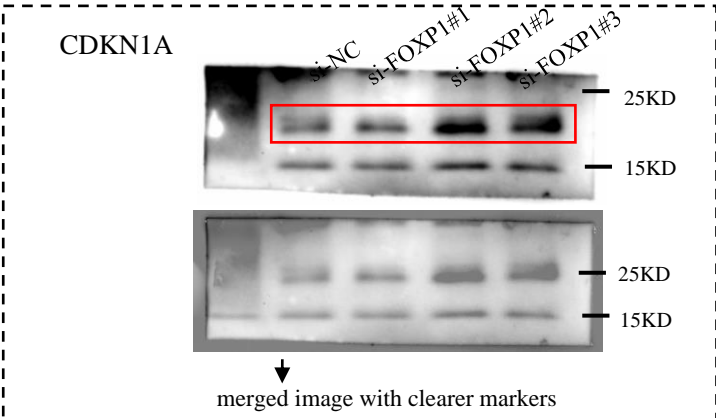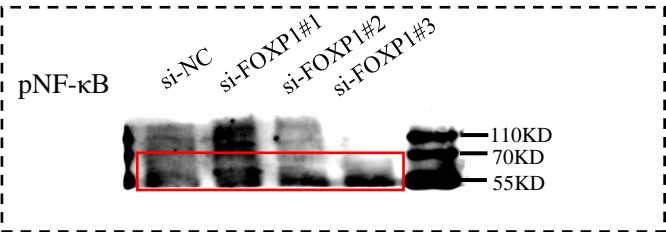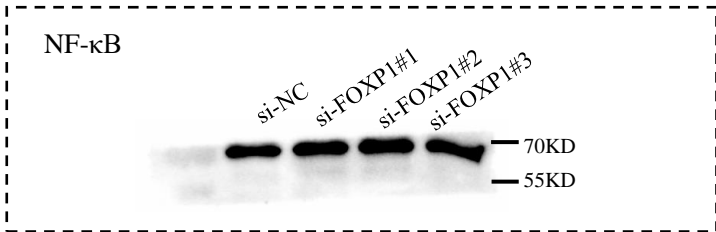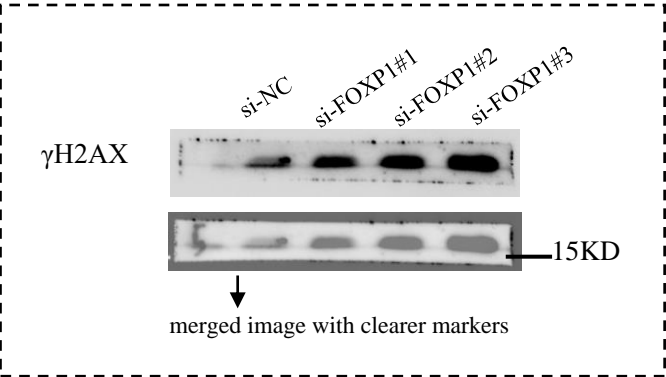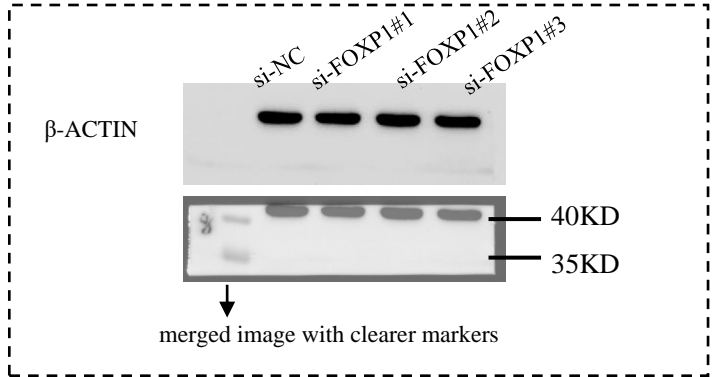

Fig. 6g pT&S

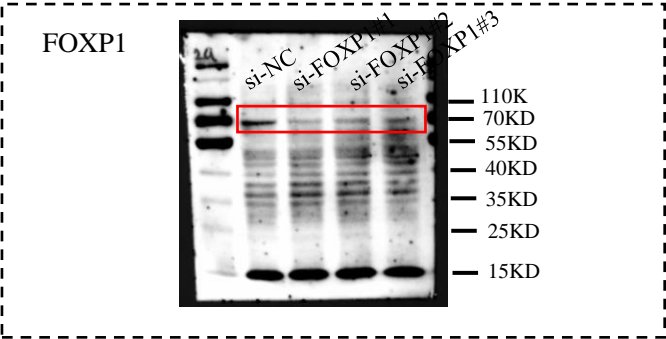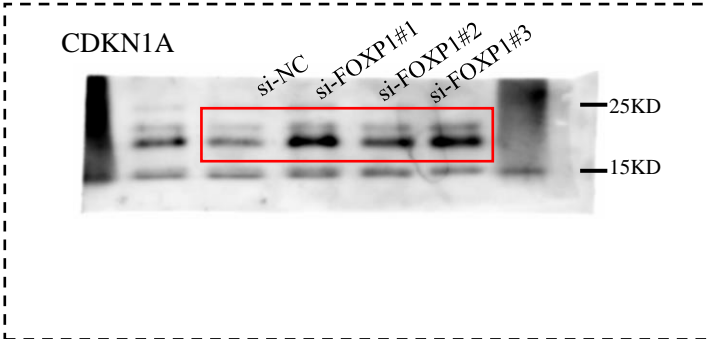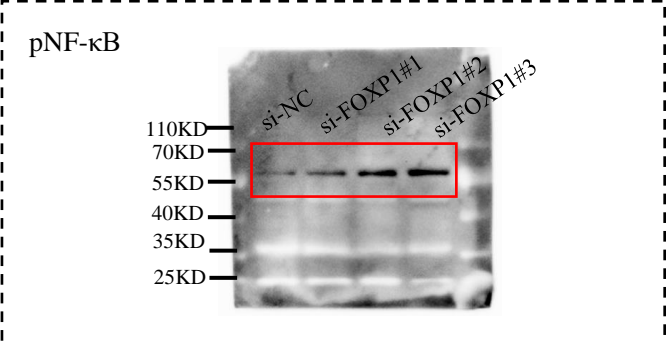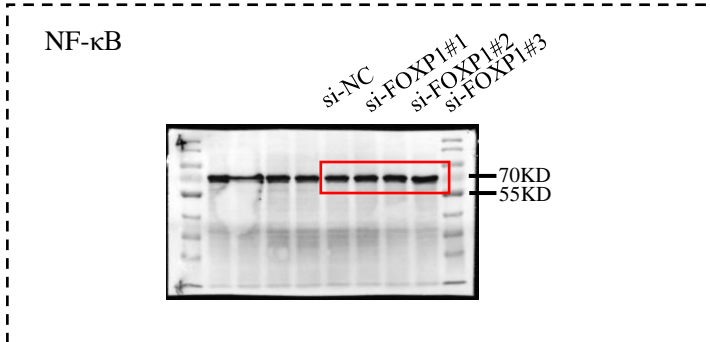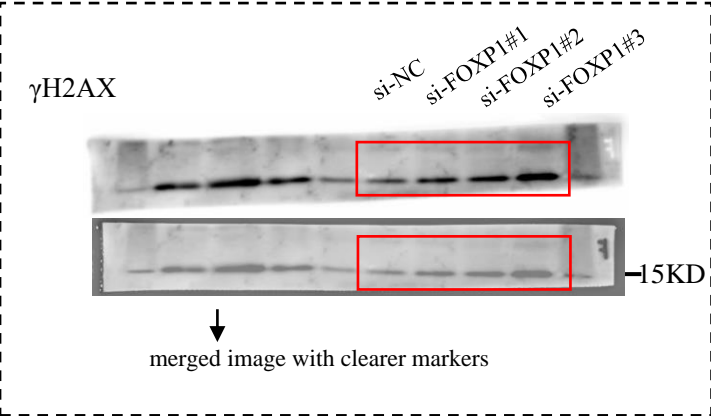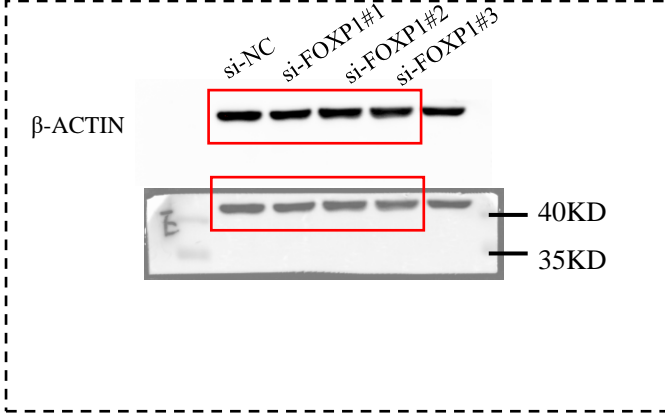

Fig. 6p

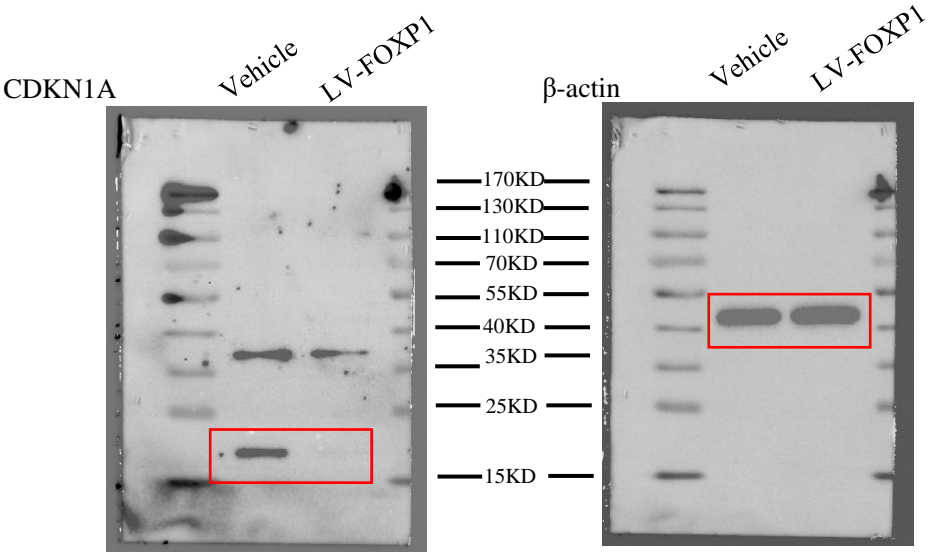

Supplement: Supplementary file 7 — Unprocessed western blots. [file 43587_2024_607_MOESM7_ESM.pdf]

Fig. 8d

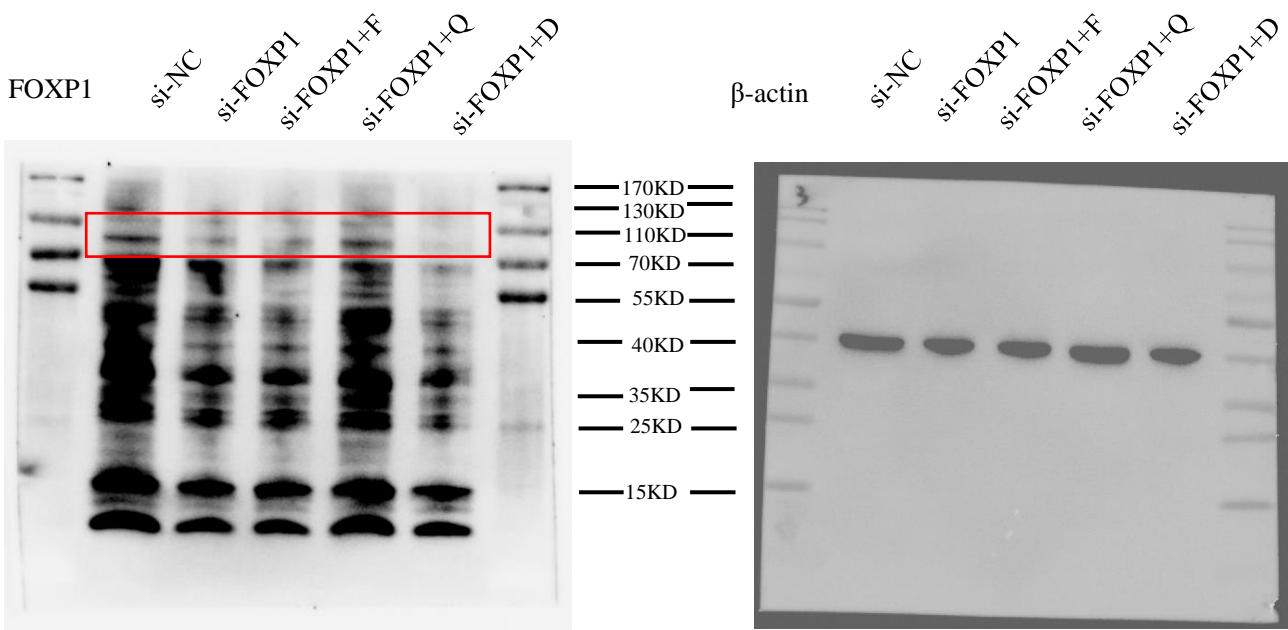

Fig. 8l

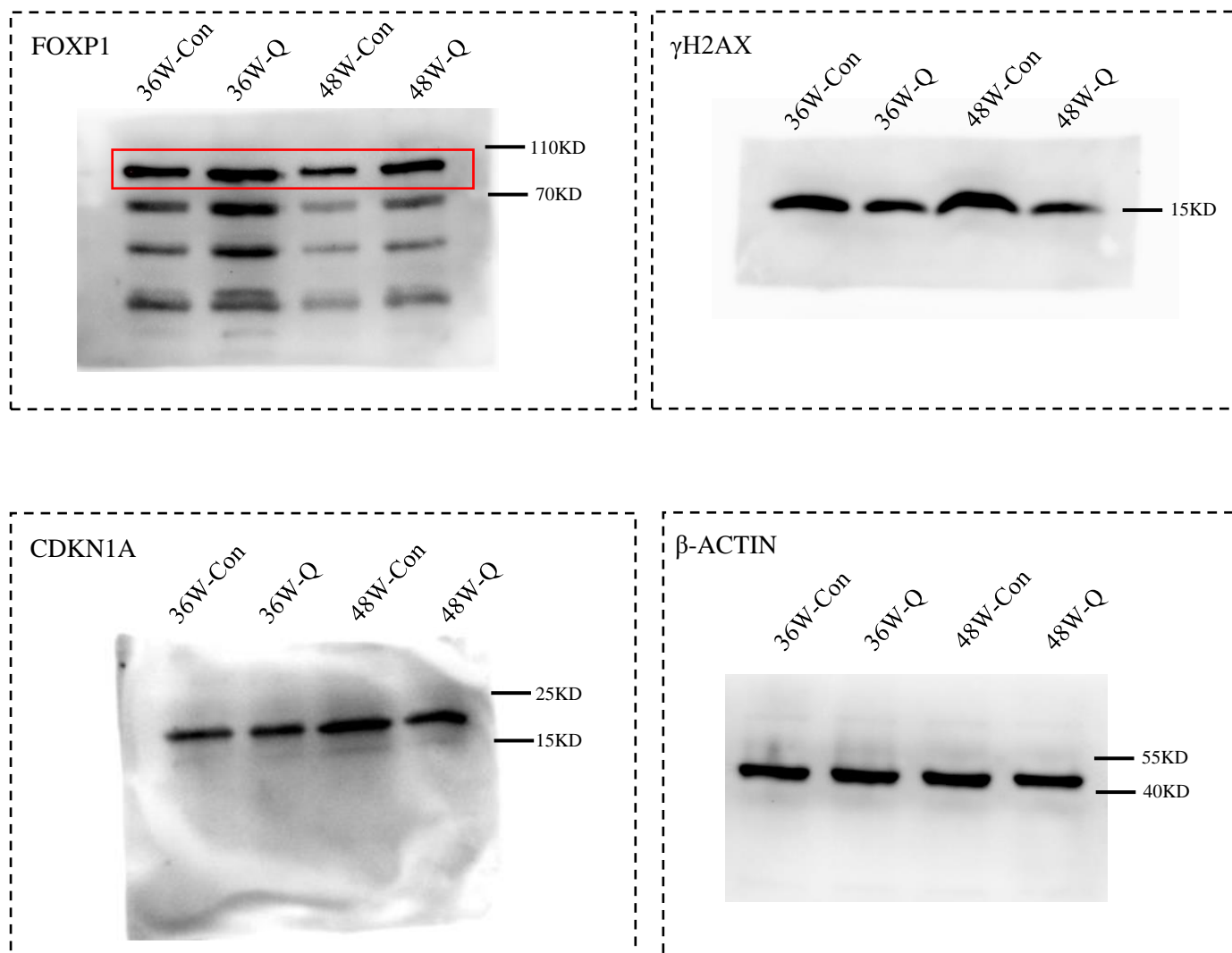

Supplement: Supplementary file 8 — Unprocessed western blots. [file 43587_2024_607_MOESM8_ESM.pdf]

Fig. S3g

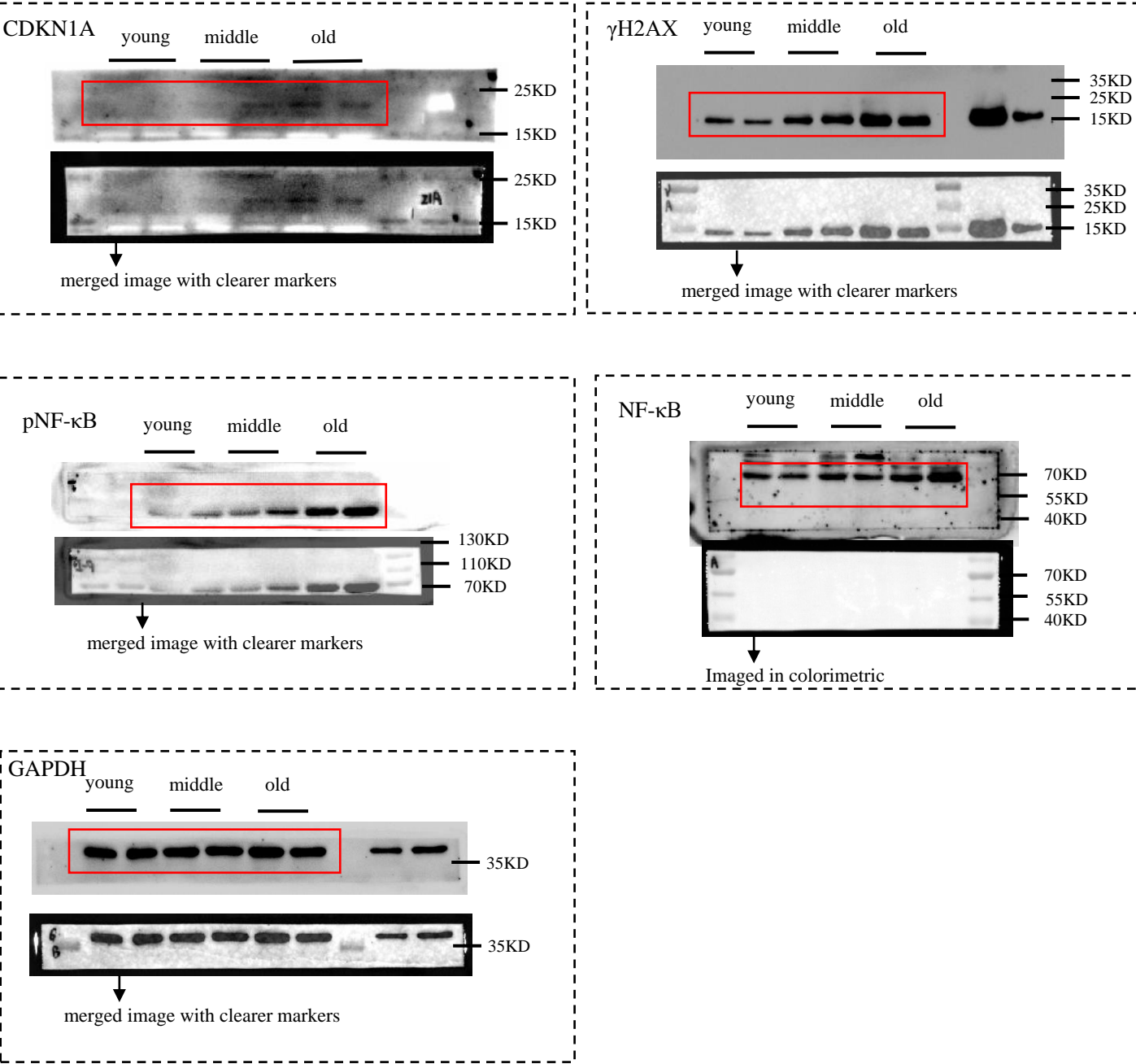

Supplement: Supplementary file 9 — Unprocessed western blots. [file 43587_2024_607_MOESM9_ESM.pdf]
